# A reference gene set construction using RNA-seq of multiple tissues of Chinese giant salamander, *Andrias davidianus*

**DOI:** 10.1093/gigascience/gix006

**Published:** 2017-02-15

**Authors:** Xiaofang Geng, Wanshun Li, Haitao Shang, Qiang Gou, Fuchun Zhang, Xiayan Zang, Benhua Zeng, Jiang Li, Ying Wang, Ji Ma, Jianlin Guo, Jianbo Jian, Bing Chen, Zhigang Qiao, Minghui Zhou, Hong Wei, Xiaodong Fang, Cunshuan Xu

**Affiliations:** 1 State Key Laboratory Cultivation Base for Cell Differentiation Regulation, College of Life Science, Henan Normal University, Xinxiang 453007, Henan Province, China; 2 Department of Laboratory Animal Science, College of Basic Medical Sciences, Third Military Medical University, Chongqing 400038, China; 3 BGI-Shenzhen, Shenzhen 518083, China; 4 Chongqing Kui Xu Biotechnology Incorporated Company, Kaixian Country, Chongqing 405423, China; 5 Xinjiang Key Laboratory of Biological Resources and Genetic Engineering, College of Life Science and Technology, Xinjiang University, Urumqi 830046, China

**Keywords:** Chinese giant salamander, *Andrias davidianus*, *De novo* transcriptome, Assembly

## Abstract

**Background:**

Chinese giant salamander (CGS) is the largest extant amphibian species in the world. Owing to its evolutionary position and four peculiar phenomenon of life (longevity, starvation tolerance, regenerative ability, and hatch without sunshine), it is an invaluable model species for research. However, lack of genomic resources leads to fewer study progresses in these fields, due to its huge genome of ∼50 GB making it extremely difficult to be assembled.

**Results:**

We reported the sequenced transcriptome of more than 20 tissues from adult CGS using Illumina Hiseq 2000 technology, and a total of 93 366 no-redundancy transcripts with a mean length of 1326 bp were obtained. We developed for the first time an efficient pipeline to construct a high-quality reference gene set of CGS and obtained 26 135 coding genes. BUSCO and homologous assessment showed that our assembly captured 70.6% of vertebrate universal single-copy orthologs, and this coding gene set had a higher proportion of completeness CDS with comparable quality of the protein sets of Tibetan frog.

**Conclusions:**

These highest quality data will provide a valuable reference gene set to the subsequent research of CGS. In addition, our strategy of de novo transcriptome assembly and protein identification is applicable to similar studies.

## Data Description

### Background

The Chinese giant salamander (CGS; *Andrias davidianus*), belonging to order Caudata, family Cryptobranchidae, is the largest extant amphibian species in the world. It is endemic to mainland China and widely distributed in central, south-western, and southern China. It is crowned as a living fossil, because it has existed for more than 350 million years [1]. It is an invaluable model species for research in the fields of evolution and phylogeny, owing to its important evolutionary position representing the transition of animals from aquatic to terrestrial life [2, 3]. However, in the past 50 years, the natural populations of CGS have sharply declined due to habitat destruction, climate change, and overhunting. This endangered amphibian has now been listed in annex I of the Convention on International Trade in Endangered Species of Wild Fauna and Flora and in class II of the national list of protected animals in China [4]. It was also listed as one of the top 10 “focal species” in 2008 by the Evolutionarily Distinct and Globally Endangered project. Natural population decline and high values for scientific conservation and medicinal use lead to its commercial aquaculture in many locations throughout China.

Despite their unique life-history characteristics, this species remains poorly characterized at the molecular level. No genomic resources are available for this species, because it has larger genomes with about 50 GB and is extremely difficult to conduct whole-genome de novo assembly even with present sequencing technology. Fortunately, RNA sequencing technologies provide cost-effective alternative approaches for the construction of the transcribed genes. Transcriptome analysis using Illumina sequencing technology has been reported in the skin and spleen of CGS [5–7], but these studies mainly discover genes associated with the immune and inflammatory response, and only two different tissues cannot obtain enough genes to research the specific biology of CGS. Here, we reported the sequenced transcriptome of more than 20 tissues from adult CGS using Illumina Hiseq 2000 technology. Our results showed that a reference gene set with high quality was constructed in this study, and it will serve as a valuable resource for future biological study of CGS.

### Samples collection

Adult female CGS with a weight of about 2 kg were obtained from an artificial breeding base, Chongqing Kui Xu Biotechnology Incorporated Company. The giant salamanders were reared in aerated, tap water-supplied tanks at 20°C and fed with diced bighead carp for 2 weeks prior to experiment. Animals were heavily anesthetized by anaesthetic MS-222 and sacrificed by dissection before sample collection. Multiple tissues (abdominal skin, dorsal skin, lateral skin, lung, heart, kidney, liver, pancreas, small intestine, spleen, stomach, brain, spinal cord, cartilage, eye, fingertip, long bone, maxillary, skull, muscle, ovary, fat, tail fat, blood) were collected.

### Sequencing

Total RNA (∼10 μg) was extracted from each sample using the TRIzol Reagent (Invitrogen). The cDNA library was contracted using TruSeq®RNA sample prep kit (Illumina) according to the manufacturer's protocol. After quality control of the cDNA libraries, pair-end sequencing was carried out via Illumina HiSeq™ 2000 at the Beijing Genomics Institute in Shenzhen. To ensure the accuracy of de novo assembly, raw reads were filtered by removal of adaptor and low-quality sequences. After preprocessing the reads, up to 156 GB of clean data were obtained in total, at least 6.4 GB of data in each sample with Q20 bases <96% (Table 1).

**Table 1:** Summary statistics of sequencing data and Q20 percentage

Samples	Clean reads	Clean data	Q20% of fq1	Q20% of fq2
Abdominal skin	71 388 238	6 424 941 420	97.86	97.15
Blood	73 523 050	6 617 074 500	97.83	96.23
Brain	72 150 562	6 493 550 580	98.10	97.24
Cartilage	72 085 300	6 487 677 000	98.00	97.33
Dorsal skin	71 852 996	6 466 769 640	97.82	96.91
Eye	72 360 422	6 512 437 980	97.96	97.44
Fat	72 747 654	6 547 288 860	97.01	95.82
Fingertip	71 793 242	6 461 391 780	98.04	97.38
Heart	71 465 342	6 431 880 780	98.00	96.71
Kidney	73 287 452	6 595 870 680	98.00	96.31
Lateral skin	71 640 046	6 447 604 140	97.95	97.30
Liver	71 772 352	6 459 511 680	98.18	96.90
Long bone	72 620 898	6 535 880 820	97.06	96.43
Lung	73 629 864	6 626 687 760	97.86	96.46
Maxillary	71 368 582	6 423 172 380	97.94	96.73
Muscle	73 184 476	6 586 602 840	97.73	96.12
Ovary	73 636 484	6 627 283 560	97.95	96.69
Pancreas	71 963 574	6 476 721 660	97.21	96.51
Skull	73 445 086	6 610 057 740	97.60	96.52
Small intestine	71 451 888	6 430 669 920	97.40	96.63
Spinal cord	72 208 398	6 498 755 820	98.14	97.30
Spleen	71 432 332	6 428 909 880	97.37	96.59
Stomach	73 740,532	6 636 647 880	97.96	96.56
Tail fat	72 435 894	6 519 230 460	98.03	97.61

### Huge RNA-seq data assembly and evaluation

**Figure 1:**
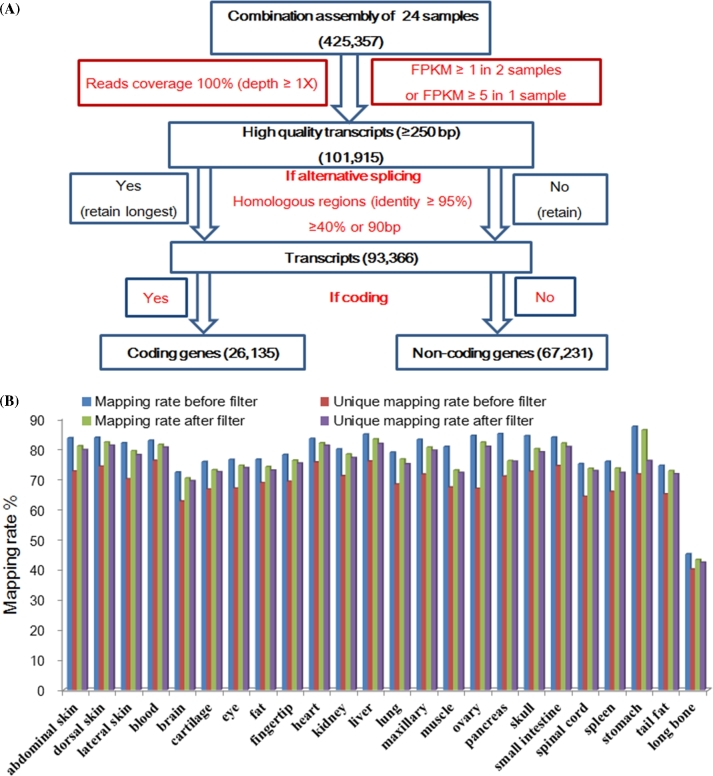
Huge RNA-seq data assembly. (A) The pipeline for de novo assembly, quality filter, and gene identification and classification. (B) The statistics of mapping rate before and after transcript filter. Among the total mapped reads, the unique mapped reads were >98% and the multiple mapped reads were <2% after transcript filter, except sample ‘stomach.’ Moreover, the total mapping rate was a slight decrease in comparison to the result before filter.

To obtain an integrated transcript set, we firstly put together all clean data and performed a combined assembly strategy by a publicly available program Trinity (V2.0.6; http://trinityrnaseq.sourceforge.net/) with the following parameters: min_kmer_cov = 3, min_glue = 3, group_pairs_distance = 250, path_reinforcement_distance = 85 [8]. It yields a huge number of transcripts, up to 425 357 transcripts output, and it includes many assembly errors and background sequences. To reduce these background and assembly errors, we developed a strict pipeline to filter these sequences (Fig. 1A). (i) Removal of assembly errors. Only each base pair in any sequence covered by at least one read was saved, except 50 bp near to the both end sequences. Any gap was trimmed, no matter where it was. The sequence was split into pieces at gap sites if there were gaps in the middle of the sequence. (ii) Removal of the background sequences. The clean reads were mapped to all the transcripts and fragments per kilobase per million mapped fragments (FPKM) value was calculated. When the expression profiling of sequence reached this standard of ≥1 FPKM in at least two samples or ≥5 FPKM in at least one sample, it was retained. (iii) Removal of isoforms produced by alternative splicing. The high homologous region (identity at least 95%) between two sequences reached this criterion: >40% or 90 bp in length of one sequence, and the shorter one was removed. (iv) Removal of short sequences. The sequence <250 bp in length was discarded. Finally, a total of 93 366 transcripts with a mean length of 1326 bp were obtained (Table 2). The clean reads were mapped to all the transcripts, and the total mapping rate and unique mapping rate ranged from 70.15% to 86.07% and 69.24% to 81.56%, respectively, except the sample ‘long bone’ (43.12% and 42.21%; Fig. 1B).

**Table 2:** The statistics of final assembly and coding gene prediction

Total data (Mb)	Total length (bp)	Total number (≥250 bp)	Total number (≥1 kp)	Average length	Coding gene	Noncoding genes
156 347	123 835 135	93 366	34 840	1326	26 135	67 231

To evaluate our filter pipeline, we compared the total mapping rate and unique mapping rate of transcripts before and after filter, respectively. After filter, the total mapping rate was slightly decreased (<2.6% average) in comparison to the total mapping rate before filter (Fig. 1B), suggesting that we retained a higher completeness rate. On the other hand, among the total mapped reads, the unique mapped reads were >98% and the multiple mapped reads were <2% after transcript filter except sample ‘stomach,’ while the multiple mapped reads were up to 10.39% (average ratio in 24 samples) before transcript filter. These data hinted that most of the redundant sequences were removed and the set of transcripts after filter had very low redundancy. Above all, our filter pipeline was effective, not only removing the assembly errors and redundancy, but also keeping most of the unique expressed sequences.

### Functional annotation

A total of 41 874 sequences can be annotated by searching against four function databases, that is, nonredundant protein database (Nr) in NCBI, Swiss-Prot, Kyoto Encyclopedia of Genes and Genomes (KEGG) pathway database, and Cluster of Orthologous Group (COG) using BLASTX (E-value ≤ 10^−5^) (Table 3). Gene ontology (GO) classification was analyzed by the Blast2GO software (v2.5.0) based on Nr annotation.

**Table 3:** Statistics for functional annotation

Functional	Number of sequences
database	annotated
NR	41 043
Swiss-Prot	30 049
KEGG	32166
COG	13 229
GO	16 369
Total	41 874

### Identification of coding gene set

**Figure 2:**
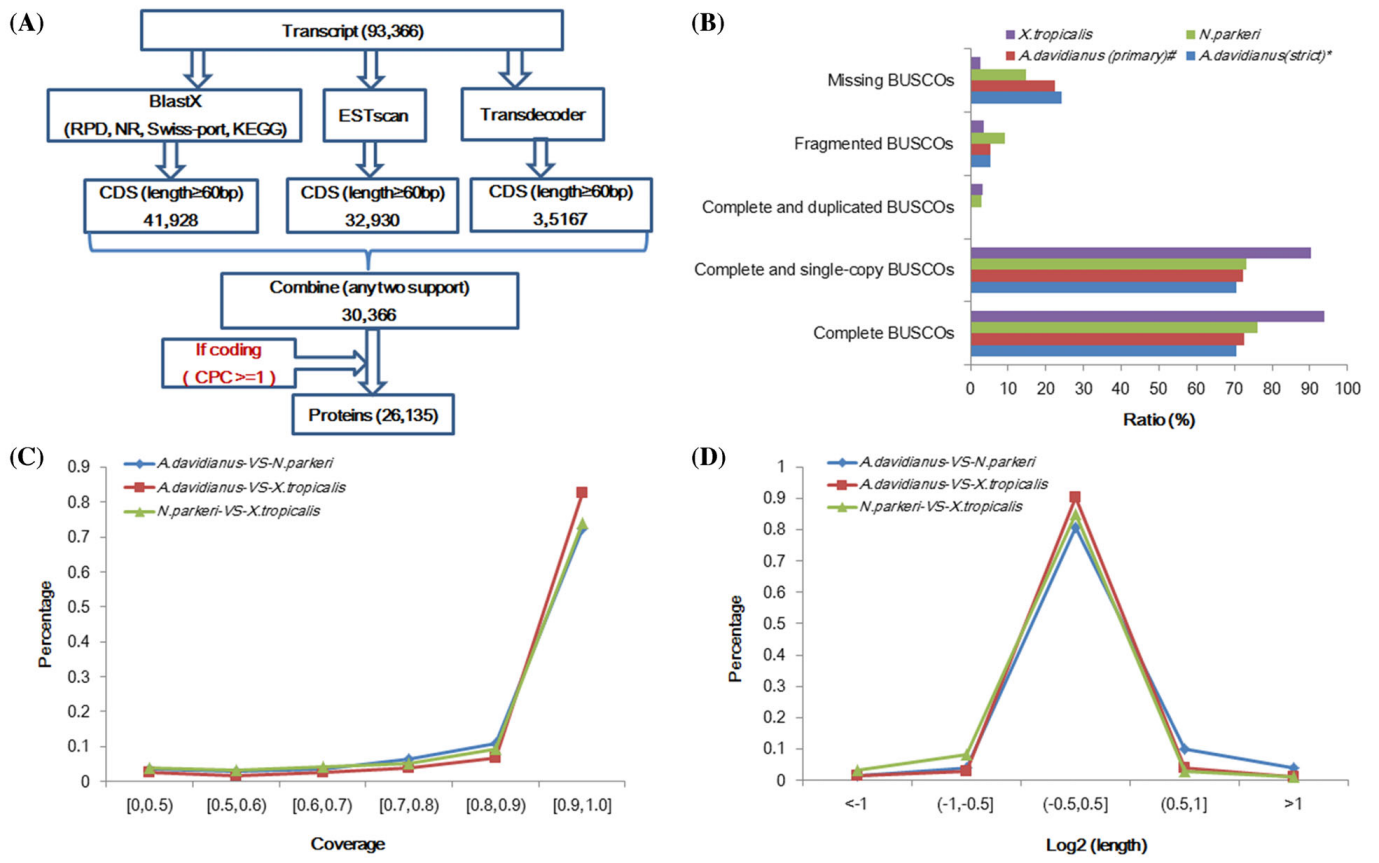
Identification and evaluation of giant salamander gene set. (A) The pipeline of prediction of coding genes. PRD represents Western clawed frog protein set, 947 proteins of CGS and 554 proteins of Newt from NCBI. (B) The results of BUSCO estimation. Asterisk (^*^) represents the final protein sets; pound (^#^) represents the primary protein sets. (C) Comparison of the length of homologous region to *X. tropicalis* and *N. parkeri* based on single copy othorlogs. The X-axis is the ratio of similarity length, and the Y-axis is the percentage of gene number at this scale. (D) Comparison of the length of CDS to *X. tropicalis* and *N. parkeri* based on single copy othorlogs. The X-axis is log base 2 of CDS length ratio, and the Y-axis is the percentage of gene number at this scale.

To identify high-quality coding genes, we developed the following pipeline to perform (Fig. 2A). Firstly, we predicted the CDS (coding sequences) of at least 60 bp using the following three methods. (i) We predicted the CDS using transdecoder (https://transdecoder.github.io/ version 2.0.1). (ii) All transcripts were searched in protein databases using BLASTX (E-value < 10^−5^) in the following order: PRD (western clawed frog protein set, 947 proteins of CGS and 554 proteins of newt from NCBI), Nr, SwissProt, and KEGG. Transcripts with sequences having matches in one database were not searched further. We selected CDS from sequences based on the best hit. (iii) All transcripts were used to predict the CDS by ESTScan (http://www.ch.embnet.org/software/ESTScan2.html; v3.0.2). Before prediction, the ESTScan was trained using the CDS data produced by BLASTX alignment method. The transcripts with CDS regions were identified by at least two methods mentioned above, and the longest CDS was chosen. Then, we filtered them with these criteria: the shortest CDS was at least 100 bp and the ratio of CDS/mRNA in length was at least >0.1. These data were defined as primary protein sets, and it was checked in the next step. Secondly, the candidate transcripts were predicted by Coding Potential Calculator software (http://cpc.cbi.pku.edu.cn/). When the transcript was reported as a coding gene and the score was ≥1, it was defined as a true coding gene. Finally, 26 135 sequences (25 965 genes after removing redundancy) passed our criterion, and they were defined as coding genes, and the remaining 67 231 sequences were defined as noncoding genes (Table 2).

### Evaluation of coding gene set

To evaluate the completeness of this coding gene set, we employed Benchmarking Universal Single-Copy Orthologs (BUSCO; http://busco.ezlab.org/) to evaluate the gene set of CGS using vertebrata data [9] and compared with two frog species, which have whole genome data available as follows: Western clawed frog (*Xenopus tropicalis*; http://ftp.ensembl.org/pub/release-81/fasta/xenopus_tropicalis/) and Tibetan frog (*Nanorana parkeri*; BioProject accession: PRJNA243398). The total number of genes for evaluation is 3023. Nearly 70.6% of total complete and single-copy BUSCOs were identified in this gene set and 73.3% (Tibetan frog) and 90.4% (Western clawed frog) of this indictor in two frogs’ gene sets (Fig. 2B). The complete and duplicated BUSCOs’ nearly zero in CGS comparing to 2.8% and 3.4% in two frogs (Fig. 2B). These data showed that our gene set had low duplicates. And the ratio of fragmented BUSCOs is 5.2%, more than Western clawed frog (3.6%) and less than Tibetan frog (9.1%) (Fig. 2B). These data hinted that we obtained an acceptable gene set of CGS, which has comparable quality of whole genome sequencing of Tibetan frog, although we only used dozens of sample by RNA-seq. We also performed the same analysis using the primary protein sets, which were only identified by three kinds of CDS prediction methods. Fortunately, these two results were much closer (70.6% and 72.3%) (Fig. 2B). These data suggest that the Coding Potential Calculator method is highly effective to remove noncoding RNAs and retain the coding mRNAs.

We advanced to evaluate the completeness of a single gene using single copy genes. To identify the single copy gene families, we selected the following reference species: *Andrias davidianus*, *Xenopus tropicalis*, *Nanorana parkeri*, *Anolis carolinensis*, *Pareuchiloglanis sinensis*, *Danio rerio*, *Oryzias latipes*, and *Homo sapiens*. For comparative analysis, we used the following pipeline to cluster individual genes into gene families using TreeFam [10]. Firstly, we collected protein sequences longer than 33 amino acids from these eight species. The longest protein isoform was retained from each gene. Secondly, BLASTP was used for all the protein sequence alignments against itself with an E-value of 1E-7. After alignment, fragmental alignments for each gene pair were conjoined using Solar [11]. Thirdly, gene families were constructed. We used average distance for the hierarchical clustering algorithm, requiring the minimum edge weight (H-score) of 10 and the minimum edge density (total number of edges/theoretical number of edges) of >1/3. The result of family classification showed that CGS obtained slightly fewer gene families than two frogs (Table 4). Finally, we identified 6634 single copy genes among three amphibian species mentioned above to evaluate the completeness of single CDS. The results showed that the percentage of CGS’s CDS with at least 90% homologous regions in Western clawed frog's ortholog (>82%) was higher than Tibetan frog vs Western clawed frog (74%; Fig. 2C) and CGS vs Tibetan frog (73%; Fig. 2C). Moreover, the CDS length of CGS was closer to Western clawed frog than Tibetan frog, and they had longer CDS than Tibetan frog (Fig. 2D). Considering differences among species, these data showed that we have obtained a higher proportion of complete CDS in this gene set.

**Table 4:** The results of gene family classification

Species	Total genes	Unclustered genes	Gene families	Unique families	Average genes per family
*A. davidianus*	26 135 (25 965)^*^	6341	12 188	520	1.62
*X. tropicalis*	18 429	218	13 235	21	1.38
*N. parkeri*	22 972	2391	13 986	306	1.47
*A. carolinensis*	17 767	818	13 387	30	1.27
*P. sinensis*	18 164	638	13 548	31	1.29
*D. rerio*	26 046	1453	13 832	177	1.78
*O. latipes*	19 671	1461	12 437	138	1.46
*H. sapiens*	21 375	2062	15 542	409	1.24

Asterisk (^*^) represents gene number after correction.

### Estimation of gene expression

**Figure 3:**
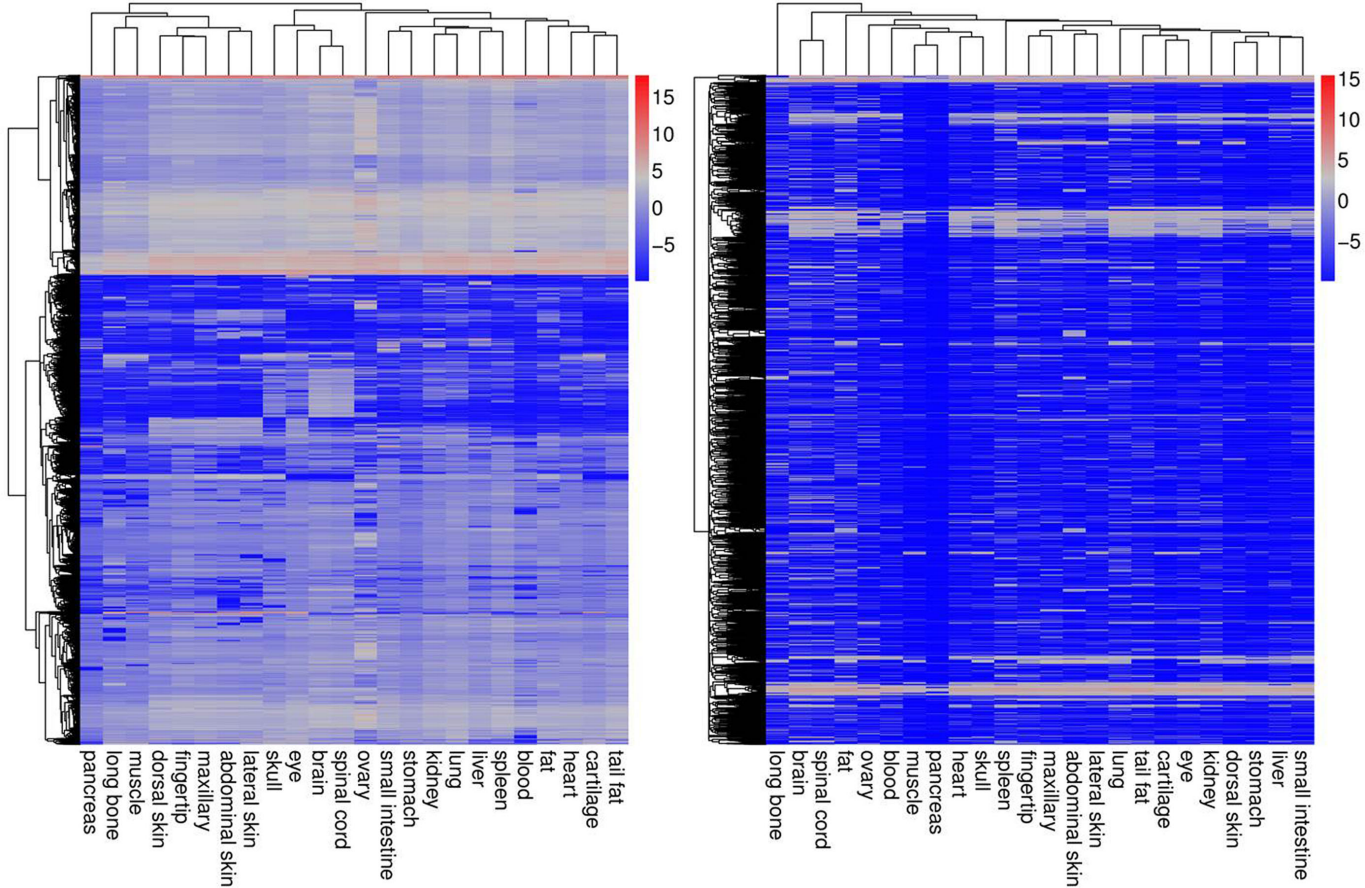
Hierarchical clustering of gene expression profiling. Coding genes (left); noncoding genes (right). The coding genes had higher expression abundances than noncoding genes.

For expression level, the clean reads of each sample were mapped to all transcripts using the Bowtie2 (version 2.2.5) software [12], then we used RSEM (v1.2.12) [13] to count the number of mapped reads and estimate FPKM values [14]. The expressed transcripts ranged from 47.32% to 75.12% of 93 366 total transcripts in each library. The expressed transcripts and genes number was illuminated in Table 5 and detail profiling of all genes was summarized in Additional file 1. The hierarchical clustering of gene expression profiling was analyzed, and the results showed that the coding genes had higher expression levels than noncoding genes (Fig. 3).

**Table 5:** The statistics of transcripts and coding genes expressed in each sample

Samples	Expressed transcripts	Coding genes	Samples	Expressed transcripts	Coding genes
Abdominal skin	53 324	20 193	Long bone	56 286	19 754
Dorsal skin	60 446	21 580	Lung	70 132	22 991
Lateral skin	53 285	20 437	Maxillary	59 431	21 424
Blood	56 540	19 994	Muscle	49 582	19 968
Brain	66 923	22 715	Ovary	53 343	21 072
Cartilage	59 724	20 979	Pancreas	44 177	18 746
Eye	67 769	22 826	Skull	59 933	22 206
Fat	65 586	21 570	Small intestine	59 156	21 588
Fingertip	63 582	21 626	Spinal cord	64 808	22 423
Heart	62 127	21 734	Spleen	64 258	21 699
Kidney	66 223	22 792	Stomach	58 688	21 601
Liver	59 755	21 622	Tail fat	63 090	21 264

### Conclusions and future directions

In summary, we sequenced 24 samples from adult CGS to construct a good reference gene set in this study, due to CGS with a huge genome size of ∼50 GB, which was hardly constructed well by present sequencing technology. A total of 26 135 coding genes with comparable quality of protein sets of Tibetan frog were identified; CGS had more coding genes than Western clawed frog with 18 429 proteins and Tibetan frog with 22 972 proteins. Moreover, this coding gene set contained approximately 70% of universal single-copy orthologs of vertebrata genes and had a higher proportion of completeness CDS with quality metrics comparable to gene set of Tibetan frog. Gene families obtained in CGS were slightly less than two frog species. Hence, we believe that CGS has more gene copies than Western clawed frog and Tibetan frog. The most likely is that more gene copies were produced with transposon element expansion and low loss rate. Sun et al. [15, 16] reported that LTR retrotransposon expansion contributed to genomic gigantism of several salamanders. A similar mechanism may contribute to CGS’s huge genome size. Obviously, we missed parts of genes, due to the fact that we sequenced 24 samples only from adult CGS. Data from other developmental stages need to be supplemented in the future study. On the other hand, the present gene sets may include some noncoding genes or other noises, even if we used the most strict pipeline to identify the proteins. This is the puzzle of RNA-seq data to identify coding genes. It needs to be verified by other data, e.g. full-length transcripts and protein data, which ought to be produced in the future. In addition, our strategy of de novo transcriptome assembly and protein identification is highly effective, and it is applicable to a wide range of other similar studies.

### Availability of supporting data

All the clean reads were deposited in the National Center for Biotechnology Information and can be accessed in the Short Read Archive (SRA accession: SRP092015) linking to BioProject accession number PRJNA350354. The assemblies and annotations data and other relevant data have also been hosted in the *GigaScience* repository, GigaDB [17].

### Competing interests

The authors declare that they have no competing interests.

### Author contributions

CSX, XDF, and HW conceived the study and designed the experiments. HTS, QG, XYZ, and JLG performed the experiments. WSL, XFG, JL, and JBJ analyzed the data. BHZ, YW, BC, ZGQ, MHZ, FCZ, JM, and JBJ contributed reagents/materials/analysis tools. XFG and WSL wrote the manuscript with input from all authors. CSX, HW, FCZ, and JM revised the paper. All authors read and approved the final manuscript.

### Additional files

Additional file 1: The expression profiling of all genes.

## Supplementary Material

GIGA-D-16-00117_Original_Submission.pdf

GIGA-D-16-00117_Revision_1.pdf

Response_to_Reviewer_Comment_Original_Submission.pdf

Reviewer_1_Report_(Original_Submission).pdf

Reviewer_1_Report_(Revision_1).pdf

Reviewer_2_Report_(Original_Submission).pdf

Reviewer_2_Report_(Revision_1).pdf

Additional File
