# Supplementary material for: A reference gene set construction using RNA-seq of multiple tissues of Chinese giant salamander, Andrias davidianus
Source: Gigascience. 2017 Feb 15;6(3):1–7. doi: 10.1093/gigascience/gix006 (PMC5467019; doi:10.1093/gigascience/gix006)
Supplement: GIGA-D-16-00117_Original_Submission.pdf [file gix006_giga-d-16-00117_original_submission.pdf]

Manuscript Click here to download Manuscript Manuscript.doc

A reference gene set construction using RNA-seq of multiple tissues provides insight into longevity, starvation tolerance and skin functions of Chinese Giant Salamander (*Andrias davidianus*)

Xiaofang Geng[1,5¶], Wanshun Li[3¶], Haitao Shang[2¶], Qiang Gou[4], Fuchun Zhang[5], Xiayan Zang[1], Benhua Zeng[2], Jiang Li[3], Ying Wang[4], Ji Ma[5], Jianlin Guo[1], Jianbo Jian[3], Bing Chen[4], Zhigang Qiao[1], Minghui Zhou[4], Hong Wei[2]*, Xiaodong Fang[3]*, Cunshuan Xu[1]*

[1] State Key Laboratory Cultivation Base for Cell Differentiation Regulation, College of Life Science, Henan Normal University, Xinxiang, China

[2] Department of Laboratory Animal Science, College of Basic Medical Sciences, Third Military Medical University, Chongqing, China

[3] BGI-Shenzhen, Shenzhen, China

[4] Chongqing Kui Xu Biotechnology Incorporated Company, Kaixian Country, Chongqing, China

[5] Xinjiang Key Laboratory of Biological Resources and Genetic Engineering, College of Life Science and Technology, Xinjiang University, Urumqi, China

* Corresponding author

E-mail: cellkeylab@126.com (CSX); fangxd@genomics.cn (XDF); weihong63528@163.com (HW)

[¶]These authors contributed equally to this work.

Abstract

Background: The Chinese giant salamander (CGS) is a valuable model species for research in the field of longevity and starvation tolerance, and its skin secretion is valuable material in traditional Chinese medicine. However, lack of genomic resources leads to fewer study progresses in these field, due to its huge genome of ~50 GB extremely difficult to be sequenced.

Results: A total of 93,366 no redundancy transcripts were obtained by RNA sequencing of twenty-four samples with a mean length of 1,326 bp. We for the first time developed two efficient pipelines to construct a high quality reference gene set of CGS and obtained 26,135 coding genes. This coding gene set had a higher proportion of completeness CDS with comparable quality of the protein sets of Tibetan frog. For longevity and starvation tolerance, we identified dozens of CGS-specific genes, several expanded proteins and three expanded signaling pathways (JAK-STAT, HIF-1 and FoxO) in comparison to the two frog species. These data will provide the first information of longevity and starvation tolerance in CGS. Meantime, we for the first time analyzed and compared the functions of three different parts of skin tissue. Interestingly, lateral skin may focus more on water and electrolyte secretion, dorsal skin on immunity and melanogenesis, and abdominal skin on water and salt metabolism.

Conclusions: These highest quality data will provide valuable reference gene set to the subsequent research of CGS. In addition, our strategy of de novo transcriptome assembly and protein identification is applicable to similar studies.

Keywords: Chinese Giant Salamander; De novo transcriptome; Longevity; Starvation tolerance; Abdominal skin; Dorsal skin; Lateral skin

Background

The Chinese giant salamander (CGS; *Andrias davidianus*), belonging to order Caudata, family Cryptobranchidae, is the largest extant amphibian species in the world. It is endemic to mainland China and widely distributed in central, south-western and southern China [1]. It is crowned as a living fossil because it has existed for more than 350 million years [2]. It is an invaluable model species for research in the fields of evolution and phylogeny, owing to its important evolutionary position representing the transition of animal from aquatic to terrestrial life [3, 4]. However, in the past 50 years, the natural populations of CGS have sharply declined due to habitat destruction, climate change and overhunting. This endangered amphibian has now been listed in annex I of the Convention on International Trade in Endangered Species of Wild Fauna and Flora (CITES) and in class II of the national list of protected animals in China [5]. It has also been listed as one of the top 10 "focal species" in 2008 by the Evolutionarily Distinct and Globally Endangered (EDGE) project. Natural population decline and high values for scientific conservation and medicinal use lead to its commercial aquaculture in many locations throughout China.

Amphibian skin that directly contacts with the external environment evolves complex structures and adaptive functions. For example, amphibian skin participates in assistant respiration [6], and has developed abundant mucous and granular glands that secrete various bioactive substances such as mucins and antimicrobial peptides to

protect against microorganisms [7]. Interestingly, giant salamander has four peculiar phenomenon of life: longevity, starvation tolerance, regenerative ability, and hatch without sunshine. Despite their unique life-history characteristics, this species remains poorly characterized at the molecular level. No genomic resources are available for this species, because it has larger genomes with about 50 GB and is extremely difficult to conduct whole-genome *de novo* assembly even with present sequencing technology. Fortunately, RNA sequencing technologies provide cost-effective alternative approaches for the construction of the transcribed gene. Transcriptome analysis using Illumina sequencing technology has been reported in the skin and spleen of CGS [8-10], but these studies mainly discover genes associated with the immune and inflammatory response, and only two different tissues can't obtain enough genes to research specific biology of CGS. Here, we reported the sequenced transcriptome of more than twenty tissues from adulthood of CGS using Illumina Hiseq 2000 technology. Our results showed that a reference gene set with high quality was constructed in this study, and it will serve as a valuable resource for future biology study. Based on this gene set, we firstly reported the molecular functions among three different parts of skin tissue and amount of specific and expansion genes relative to longevity and starvation tolerance in comparison to other two amphibian species.

Results and Discussion

Huge RNA-seq data assembly

To maximize information on the genes of the Chinese Giant Salamander (CGS),

we collected twenty-four samples from different tissues, and each sample was sequenced in separate library. After the preprocessing of reads, up to 156 GB of clean data were obtained in total, at least 6.4 GB of data in each sample with Q20 bases more than 96 % (Additional file 1). To obtain an integrated transcript set, all data were subjected for de novo transcriptome assembly by a publicly available program Trinity [11]. It yields a huge transcript data, up to 425,357 transcripts output, and it includes lots of assembly errors and background sequences. To eliminate them, we developed a strict pipeline to filter these sequences (Fig. 1A), due to that twenty-four samples analyzed in this study belong to different tissues except skin tissues, containing almost all coding genes in adulthood. Finally, a total of 93,366 transcripts (more than 250 bp) with a mean length of 1,326 bp were used for advanced analysis (Table 1). The clean reads were mapped to all the transcripts, and the total mapping rate and unique mapping rate ranged from 70.15-86.07 % and 69.24-81.56 %, respectively, except sample 'long bone' (43.12 % and 42.21 %; Fig. 1B). Compared to total mapping rate, the unique mapping rate was less than 2 % in twenty-three samples, except sample 'stomach'. This data hinted that the set of transcripts had very low redundancy. On other hand, the total mapping rate was a slight decrease in comparison to the result before filter (Fig. 1B). These data showed that our filter pipeline made a good effective, not only removing the assembly error and redundancy, but also keeping all the unique expressed sequences. The expressed transcripts ranged from 47.32 % to 75.12 % of 93,366 total transcripts in each library (Table 2).

Identification and evaluation of giant salamander gene set

To identify high-quality coding proteins, we developed a strict pipeline to scan coding sequences (Fig. 2A). At last, 26,135 sequences (25,965 genes after removing redundancy) were passed our criterion, which were defined as coding genes, and the rest of 67,231 sequences were defined as non-coding genes (Table 2). The profiling of all genes was summarized in Additional file 2. The hierarchical clustering of gene expression profiling was analyzed, and the results showed that the coding genes had higher expression level than non-coding genes (Additional file 3). Subsequently, we employed BUSCO (Benchmarking Universal Single-Copy Orthologs; http://busco.ezlab.org/) [12] method to estimate the completeness of this coding gene set on CGS (*Andrias davidianus*), and compared it with the two frog species Western clawed frog (*Xenopus tropicalis*) and Tibetan frog (*Nanorana parkeri*). The total number of genes for evaluation is 3023. Nearly 70.6 % of total complete and single-copy BUSCOs were identified in this gene set and 73.3 % (Tibetan frog) and 90.4% (Western clawed frog) of this indictor in two frogs' gene sets (Fig. 2B). The 'Complete and duplicated BUSCOs' nearly zero in CGS compare to 2.8% and 3.4% in two frogs (Fig. 2B). This data showed that our gene set had low duplicates. And the ratio of 'Fragmented BUSCOs' is 5.2%, more than Western clawed frog (3.6%) and less than Tibetan frog (9.1%) (Fig. 2B). These data hinted that we obtained an acceptable gene set of CGS which has comparable quality of whole genome sequencing of Tibetan frog, although we only used dozens of sample by RNA-seq. We also performed the same analysis using the primary protein sets, which were only identified by three kinds of CDS prediction methods (see Methods). Fortunately, these

two results were very similar (70.6 % and 72.3 %) (Fig. 2B). These data suggest that CPC (Coding Potential Calculator) method has highly effective to remove non-coding RNAs and remain the coding mRNAs. We also used 6,634 single copy genes among three species mentioned above to evaluate the completeness of single CDS. The results showed that the percentage of CGS's CDS with at least 90 % homologous regions in Western clawed frog's ortholog (more than 82 %) was higher than Tibetan frog-vs-Western clawed frog (74%; Fig. 2C) and CGS-vs-Tibetan frog (73%; Fig. 2C). Moreover, the CDS length of CGS was closer to Western clawed frog than Tibetan frog, and they had longer CDS than Tibetan frog (Fig. 2D). Considering differences among species, this data showed that we have obtained a higher proportion of completeness CDS in this gene set.

Analysis of gene families and Chinese giant salamander-specific genes

Using TreeFam method (see Methods), we obtained 12,188 gene families, 520 unique families and 6,341 un-clustered genes from CGS and other seven reference genomes (Table 3). The total gene families were slightly less than other species, and unique families were more than other species. The common and unique gene families among *A. davidianus*, *X. tropicalis*, *N. parkeri* and *H. sapiens* are summarized in Additional file 4.

To further understand the CGS-specific gene set, we carried out an analysis to eliminate the homologous genes of relative species (see Methods). At last, 3,839 proteins were remained, and then annotated by KEGG pathway according to the best hit (Additional file 5). The results showed that most of the enriched pathways were

associated with immune and inflammatory response. Interestingly, JAK-STAT and HIF-1 signaling pathways, as important pathways related to longevity, were significantly enriched by CGS-specific genes. In addition, pathways associated with longevity and starvation tolerance, such as insulin and PI3K-Akt signaling pathways, were enriched by KEGG pathway analysis. Moreover, several other longevity-related pathways such as Ras, MAPK and NF-kappa B signaling pathways, and starvation tolerance-related pathways such as calcium signaling pathway, regulation of lipolysis in adipocytes and glycolysis/ gluconeogenesis were also enriched.

Longevity

Chinese giant salamander can survive up to 200 years, and has various mechanisms to expand life span. In this study, to understand the mechanism of longevity, we found the following two differences between CGS and Western clawed frog or Tibetan frog. Firstly, we identified 23 CGS-specific genes in several pathways associated with longevity using a strict method (see Methods; Fig. 3A and Additional file 6). These genes only existed in CGS in theory. Ten and eleven CGS-specific genes were significantly enriched in HIF-1 signaling pathway and JAK-STAT signaling pathway, respectively, which were positive to longevity. More other pathways regulated longevity including PI3K-Akt signaling pathway (6 genes), NF-kappa B signaling pathway (3 genes), ras signaling pathway (2 genes) and insulin signaling pathway (1 gene). Secondly, HIF-1, JAK-STAT and FoxO signaling pathways were expanded to other two amphibians (Fig. 3A and Additional file 6). Insulin/IGF-1 signaling pathway, as evolutionarily well-conserved pathway, has been shown to

negatively regulate longevity in many organisms, ranging from simple invertebrates to mammals [13, 14]. PI3K-Akt pathway and Ras pathway, as two branches of insulin/IGF-1 pathway were also reported to regulate longevity. For example, both decreased PI3K (AGE-1) activity and increased PTEN (DAF-18) expression result in extended longevity of *Caenorhabditis elegans* [15]. Ras signaling pathway in higher eukaryotes negatively regulates longevity, and direct reduction of Ras activity leads to increased lifespan [16, 17]. AMPK and HIF-1 control immunity and longevity tightly by acting as feedback regulators of ROS in response to reduced mitochondrial respiration [18]. We advanced analysis showed that IFNGR2 (K05133), AKT (K04456), PI3K (K00922), IL20RA (K05136), IL8 (K10030), PRKAR2 (K04739), PLA2 (K16342), GAB1 (K09593), SRF (K04378) and USP7 (K11838) have more gene copies than other two amphibians (Western clawed frog and Tibetan frog). A study showed that elevated expression of the IFN-γ receptor protein IFNGR2 was found in long-lived primate species [19], which verified our result that IFNGR2 with more gene copies in CGS contributed to longevity. USP7 was expanded in CGS and played an integral role in mechanisms regulating healthspan and lifespan extension by controlling the stability of DAF-16/FOXOs as contributors for extreme longevity [20]. Therefore, we speculate that JAK-STAT, HIF-1, FoxO and insulin signaling pathways might be the important pathways to control longevity of CGS by regulating the expression of key genes such as IFNGR2, PI3K and AKT.

Starvation tolerance

Giant salamander has strong starvation tolerance. Under suitable conditions,

adult giant salamanders can survive two years without eating. To understand the mechanism of starvation tolerance, we found several differences between CGS and Western clawed frog or Tibetan frog. Firstly, we identified 11 CGS-specific genes in several pathways associated with starvation tolerance using a strict method (see Methods; Fig. 3B and Additional file 6). Pathways associated with starvation tolerance include PI3K-Akt signaling (6 genes), glycolysis/gluconeogenesis (2 genes), regulation of lipolysis in adipocytes (1 gene), calcium signaling (1 gene) and insulin signaling (1 gene) (Fig. 3B and Additional file 6). In adipocytes, the hydrolysis of triacylglycerol to produce fatty acids and glycerol under fasting conditions is tightly regulated by neuroendocrine signals, resulting in the activation of lipolytic enzymes [21]. Calcium-mediated signaling pathway regulated FoxO1 nuclear localization and hepatic glucose homeostasis during fasting [22]. We advanced analysis showed that AKT (K04456), PI3K (K00922), PRKAR2 (K04739), ATP2B (K05850), RYR2 (K04962) and USP7 (K11838) had more gene copies than the other two amphibians (Western clawed frog and Tibetan frog). USP7 suppresses the fasting/cAMP-induced activation of gluconeogenic genes in liver depending on FoxO1, resulting in decreased hepatic glucose production [23]. Therefore, we speculate that glycolysis/gluconeogenesis and fatty acid oxidation might play important roles in starvation tolerance of CGS through the expansion of key genes.

Characteristic of each tissue

In order to characterize the relationship among the 24 kinds of sample, clustering was used in this study. The results showed that these tissues were categorized into 7

clusters (Additional file 7). Obviously, brain and spinal cord were in one cluster, and skull, cartilage and eye in another cluster. Interestingly, lateral skin, abdominal skin, dorsal skin, fingertip, and maxillary were classified into a cluster. This could be because fingertip and maxillary contain skin tissues in sample preparation.

Our network of co-expression modules (gene clusters) detects tissue-specific differences. We assume that co-expressed genes have similar functions [24]. This approach helps to identify the functions of un-annotated genes by correlating them with annotated genes. Our analysis clusters 26,135 unigenes into 24 co-expressed modules and we denote the modules using different colors in Fig. 4. Generally, modules contain unigenes enriched for specific biological functions, and this provides evidence of the molecular functions of un-annotated unigenes. Correlations between modules and tissues exist, and we mapped the unigenes in each module against the KEGG pathway. For example, black module strongly correlates with the skin tissue, and highly expressed proteins in black module were significantly enriched in immune system pathways, melanogenesis-related pathways and metabolic pathways, consistent with the biological function of skin which likely functions in first-line defense processes. Brown strongly correlates with brain and spinal cord, and highly expressed unigenes in brown module were significantly enriched in glutamatergic synapse, GABAergic synapse and neuroactive ligand-receptor interaction, consistent with the biological function of brain and spinal cord as the core of the nervous system. Brown4 strongly correlates with the liver tissue. As expected, complement and coagulation cascades, phagosome, bile secretion and metabolic pathways were

significantly enriched in the liver tissue. Darkgreen strongly correlates with small intestine, and highly expressed unigenes were significantly enriched in the digestion and absorption of vitamin, fat protein and carbohydrate, which were consistent with the biological function of small intestine. Darkgrey and lightgreen strongly correlate with the ovary tissue. The highly expressed unigenes of ovary were enriched in protein synthesis, DNA replication, oocyte meiosis and regulation of autophagy. Floralwhite strongly correlates with the kidney, which can eliminate nitrogen-containing metabolites. Highly expressed unigenes in floralwhite module were significantly enriched in protein metabolic pathways, such as lysine biosynthesis, arginine and proline metabolism, and histidine metabolism. Plum2 strongly correlates with the eye tissue. Phototransduction was significantly enriched in the highly expressed unigenes of eye. These results will aid in the annotation of the CGS genome.

Comparison of the function of three different parts of skin tissue

Amphibian skin that directly contacts with the external environment evolves complex structures and adaptive functions such as assistant respiration and abundant mucous and granular glands, since the amphibian represents the transitional vertebrate from aquatic to terrestrial life. Presently, transcriptome [9] and proteomics [25, 26] analysis of the skin of CGS have been reported. However, the molecular functions of three different parts of skin tissue (dorsal, abdominal and lateral skin) of CGS are still unclear. To investigate whether differences exist in the function of three different parts of skin tissue of CGS, we further analyzed the 797 genes in the black module. The

main functions of genes in the black module by co-expression network analysis were biosynthesis, melanin, immunity, metabolism, and secretion (Fig. 5A-E). Then, we detected the tissue-specific expressed genes among the three different parts of skin tissue of CGS (see Methods), and identified 139 specifically expressed genes in the abdominal skin, 127 genes in the dorsal skin, and 108 genes in the lateral skin, which could be seen in Additional file 8.

There were 81 hub genes in the black module identified by WGCAN (Additional file 9). Among these hub genes, several genes such as desmoglein-4 (DSG4), aquaporin 5 (AQP5), epithelial chloride channel protein, myeloperoxidase (MPO) and mucin-2 (MUC2) were higher expressed in the lateral skin. DSG4 plays a role in cell-cell adhesion in epithelial cells [27]. Water channel protein AQP5 and epithelial chloride channel protein play roles in the generation of secretions and iron transport [28, 29]. MPO and MUC2 as skin mucus defense molecules regulate defense response [30, 31]. In addition, KEGG pathway analysis based on tissue-specific expressed genes showed that tight junction was only significantly enriched by lateral skin. Meantime, the sum of gene expression quantity of vibrio cholerae infection which regulates ion transport, water and electrolyte secretion and tight junctions in the lateral skin was higher than that in the abdominal and dorsal skin. Therefore, we speculate that lateral skin of CGS may focus more on water and electrolyte secretion, ion transport and tight junction compared to abdominal skin and dorsal skin.

Meantime, several other hub genes such as corticosteroid 11-beta-dehydrogenase isozyme 1 (HSD11B1) and patatin-like phospholipase domain-containing protein 1

(PNPLA1) were higher expressed in the abdominal skin. HSD11B1 plays a role in glucocorticoid metabolism which could regulate water and salt metabolism and inflammatory response [32, 33]. It has been shown that PNPLA1 exists in the epidermis and stronger expression in the granular layer, and has a role in glycerophospholipid metabolism in the cutaneous barrier [34]. Furthermore, metabolic pathways were significantly enriched by the abdominal skin, and the sum of gene expression quantity of metabolic pathways, alanine, aspartate and glutamate metabolism, and mineral absorption in the abdominal skin was higher than that in the lateral skin (Fig. 5F), indicating that abdominal skin of CGS may focus more on water and salt metabolism.

Tyrosinase was higher expressed in the dorsal skin, which plays essential roles in the melanogenesis. Moreover, melanogenesis and tyrosine metabolism were significantly enriched in dorsal skin by KEGG pathway analysis, and the sum of gene expression quantity of melanogenesis, phenylalanine metabolism, tyrosine metabolism and metabolic pathways in the dorsal skin was higher than that in the abdominal skin and lateral skin (Fig. 5F), suggesting that melanogenesis in the dorsal skin may be stronger than that in the abdominal and lateral skin. In addition, some hub genes were both highly expressed in the dorsal and abdominal skin such as interleukin-18 receptor 1 (IL18R1), C-C motif chemokine 28 (CCL28) and cell death-inducing p53-target protein 1 homolog (LITAF), which mediated immune and inflammatory response. Further KEGG pathway analysis showed that the pathways associated with immune response, such as antigen processing and presentation and

pathogenic *Escherichia coli* infection, were all enriched by dorsal, abdominal and lateral skin. However, the sum of gene expression quantity of immune response in the dorsal skin, such as amoebiasis, pathogenic *Escherichia coli* infection, staphylococcus aureus infection and sphingolipid metabolism, was higher than that in the abdominal skin and lateral skin (Fig. 5F), indicating that the immunity of the dorsal skin may be stronger than that of the abdominal and lateral skin.

In total, based on the results of co-expression network and KEGG pathway analysis, we speculate that the lateral skin of CGS may focus more on water and electrolyte secretion, ion transport and tight junction; the dorsal skin may focus more on immunity and melanogenesis; the abdominal skin may focus more on water and salt metabolism.

Secreted proteins in skin

In response to stress or predator attack, amphibian skin secretes a complex chemical cocktail, and these secretions contain a plethora of biologically active components, including alkaloids, biogenic amines, peptides and proteins [35]. To further understand the secreted proteins of CGS, we predicted it based on signal peptide through website http://www.cbs.dtu.dk/services/SignalP/, and identified 2124 secreted proteins (Additional file 10). Among them, 17 proteins belong to antibacterial peptides (Additional file 11). Moreover, 975 proteins of the 2124 secreted proteins were found in the abdominal skin, 921 proteins in the dorsal skin and 906 proteins in the lateral skin (Additional file 8). Interestingly, analysis of tissue-specificity showed that immune response-related proteins (interleukin-20

receptor subunit beta, alpha-2-macroglobulin-like protein 1 and avidin-related protein 4/5), uroplakin-3b-like protein, and fibulin-2 were all highly expressed in the three different parts of skin tissue. Fibulin-2 is involved in extracellular matrix proteolysis or remodeling of keratinocytes [36]. Moreover, norrin and BPI fold-containing family C protein (BPIFC) were higher expressed in the lateral skin. Norrin induced endothelial cells proliferation, survival and migration by activating Wnt/β-catenin signaling [37]. BPIFC, also known as BPIL2, was detected prominently in the basal layer of the epidermis from inflammatory skin of psoriasis specimens [38]. Several secreted proteins were highly expressed in the dorsal skin including neuromedin-B, tyrosinase-related protein-1 (TYRP1), melanocyte-specific protein QNR-71, melanocyte protein PMEL, WNT1-inducible-signaling pathway protein 1 (WISP1) and aminopeptidase N. Neuromedin B is homologous to the amphibian bombesin-like peptide ranatensin isolated from Rana pipiens skin libraries, and thus may elicit behavioral effects similar to those of bombesin [39, 40]. WISP-1 inhibits melanoma growth by the activation of Notch1 signaling in stromal fibroblasts [41]. Aminopeptidase N was expressed in dermal fibroblasts by keratinocyte-derived stimuli, and could serve as a target in the regulation of MMP1 expression in epidermal-mesenchymal communication [42]. In addition, serine/arginine repetitive matrix protein 2 (SRRM2) for mRNA splicing and matrix metalloproteinase-18 (MMP18) for proteolysis were highly expressed in the abdominal skin. In short, these results once again confirmed the functions of the three different parts of skin tissue.

Conclusions

We sequenced 24 RNA-seq samples from adult of CGS to construct a good reference gene set in this study, due to CGS with a huge genome size of ~50 GB, which was hardly constructed well by present sequencing technology. A total of 26,135 coding genes with comparable quality of protein sets of Tibetan frog were identified; CGS has more gene number than Western clawed frog of 18,429 proteins and Tibetan frog of 22,972 proteins. Moreover, this coding gene set contains approximately 70 % of Universal Single-Copy Orthologs of vertebrata genes, and had a higher proportion of completeness CDS with quality metrics comparable to gene set of Tibetan frog. Gene families obtained in CGS were slightly less than the other two amphibian species. Hence, we believe that CGS has more gene number than Western clawed frog and Tibetan frog. The most likely is that more gene copies were produced with transposon element expansion and low loss rate. Sun et al. [43, 44] reported that LTR retrotransposons expansion contributed to genomic gigantism of several salamanders. Similar mechanism may contribute to CGS's huge genome size. Obviously, we missed parts of genes, due to that we only sequenced tissues in adult period. Other developmental stages need complement in future study. In other hand, the present gene sets maybe include some non-coding genes, redundant genes or other noises, even if we used a most strict pipeline to identify the proteins. This is puzzle of RNA-seq data to identify coding genes. It needs to be verified by other data, full-length transcripts and protein data which ought to produce in the future. In addition, our strategy of de novo transcriptome assembly and protein identification

works high effectively, and it is applicable to a wide range of other similar studies.

Methods

Animals and sample preparation

Adult female Chinese giant salamanders with weight of about 2 kg, were obtained from an artificial breeding base Chongqing Kui Xu Biotechnology Incorporated Company, which had obtained the permission of aquatic wild animal domestication and breeding management from the fishery administration of China. The giant salamanders were reared in aerated, tap water supplied tanks at 20 °C and fed with diced bighead carp for 2 weeks prior to experiment. Animals were heavily anesthetized by anaesthetic MS-222 and sacrificed by dissection before sample collection. Multiple tissues (abdominal skin, dorsal skin, lateral skin, lung, heart, kidney, liver, pancreas, small intestine, spleen, stomach, brain, spinal cord, cartilage, eye, fingertip, long bone, maxillary, skull, muscle, ovary, fat, tail fat, blood) were collected, flash-frozen in liquid nitrogen and stored at -80 °C until use. All experiments were performed in accordance with the guidelines of the Animal Ethics Committee and were approved by the Institutional Review Board on Bioethics and Biosafety of BGI (No. FT15103).

Sequencing and filter

Total RNA (~10 μg) was extracted from each sample using the Trizol Reagent (Invitrogen). RNA samples were subjected to DNase I digestion to remove remaining DNA. Poly-A RNA was purified and enriched using poly-T oligo-coated magnetic beads (Invitrogen). Following purification and fragmentation, first-strand cDNA was

generated using Superscript II reverse transcriptase (Invitrogen) and random hexamer primers. The cDNA was further converted into double stranded DNA using TruSeq®RNA sample prep kit (Illumina). After quality control of the cDNA libraries, pair-end sequencing analysis was carried out *via* Illumina HiSeq™ 2500 at the Beijing Genomics Institute in Shenzhen according to the Illumina manufacturer's protocol.

To ensure the accuracy of de novo assembly, raw reads were filtered by removal of adaptor and low quality sequences. The reads containing the sequencing adaptor, more than 5 % unknown nucleotides and more than 20 % bases of quality value less than 10, were eliminated. This output was termed 'clean reads'. After removal of PCR duplicate reads, the rest reads were used for assembly.

Assembly and gene set analysis

To obtain an integrated transcript set, all tissues were *de novo* assembled using a combined assembly strategy by a publicly available program Trinity (V2.0.6; http://trinityrnaseq.sourceforge.net/) with the following parameters: min_kmer_cov=3, min_glue=3, group_pairs_distance=250, path_reinforcement_distance=85, bfly_opts '-V 5 --edge-thr=0.1 --stderr' [11].

After the primer assembly, a huge number of transcripts were yielded including error and background sequences. To reduce the background and assembly errors, we developed a strict pipeline to filter these sequences. 1) Removal of assembly errors. Only each base pair in any sequence covered by at least one read will be saved, except 50 bp near each end of sequence. If there have gaps in the middle of sequence,

this sequence will be split into pieces at gap sites, and the gap will be trimed. When the gaps exist at end of sequence, these gaps will be trimmed. 2) Removal of the background sequences. The clean reads were mapped to all the transcripts and FPKM value was calculated. When the expression profiling of sequence reached this standard of ≥ 1 FPKM in at least two samples or ≥ 5 FPKM in at least one sample, it will be retained. 3) Removal of isoforms produced by alternative splice. The high homologous region (identity at least 95 %) between two sequences reaches to one of the criterion: larger than 40 % or 90 bp in length of one sequence, and the shorter one will be removed. 4) Removal of short sequences. The sequence with less than 250 bp in length will be discarded. Finally, based on the sequence similarity, the transcripts were divided into two classes: clusters (prefixed with 'CL') and singletons (prefixed with 'unigene'). In each cluster, one sequence can search a hit with matched region of at least 60 % in length, and the transcripts were homologous genes. After the above pipeline, the rest sequences as final transcript set were analyzed.

Coding proteins and secreted proteins

To identify high-quality coding proteins, we developed the following pipeline to perform. Firstly, we predicted the CDS (coding sequences) of at least 60 bp using the following three methods. 1) We predicted the CDS using transdecoder (https://transdecoder.github.io/ version 2.0.1). 2) All transcripts were searched in protein databases using blastx (E-value < 10^{-5}) in the following order: PRD [western clawed frog protein set, 947 proteins of CGS and 554 proteins of newt from NCBI],

Nr, SwissProt and KEGG. Transcripts with sequences having matches in one database were not searched further. We selected CDS from sequences based on the best hit. 3) All transcripts were used to predict the CDS by ESTScan (http://www.ch.embnet.org/software/ESTScan2.html; v3.0.2). Before prediction, the ESTScan was trained using the CDS data produced by blastx alignment method. The transcripts with CDS regions were identified by any two methods mentioned above and the longest CDS will be chosen. Then, we filtered them with these criteria: the shortest CDS was at least 100 bp and the ratio of cds/mRNA in length was at least more than 0.1. This data were defined as 'primary protein sets', and will be checked in next step. Secondly, the candidate transcripts will be predicted by CPC (Coding Potential Calculator) software (http://cpc.cbi.pku.edu.cn/). When the transcript was reported as a coding gene and the score was no less than 1, it was defined as a true coding gene. To evaluate the completeness of this coding gene set, we employed BUSCO (Benchmarking Universal Single-Copy Orthologs; http://busco.ezlab.org/) to evaluate the gene set of CGS using vertebrata data [12] and compared with other frogs, which have whole genome data available as follows, Western clawed frog (*Xenopus tropicalis*; http://ftp.ensembl.org/pub/release-81/fasta/xenopus_tropicalis/) and Tibetan frog (*Nanorana parkeri*; BioProject accession: PRJNA243398). The secreted protein was predicted by website http://www.cbs.dtu.dk/services/SignalP/.

Comparative and evolutionary analysis of gene family

To identify the gene families and CGS-specific genes, we selected the following reference species: *A. davidianus*, *X. tropicalis*, *N. parkeri*, *A. carolinensis*, *P. sinensis*,

D. rerio, *O. latipes* and *H. sapiens*. For comparative analysis, we used the following pipeline to cluster individual genes into gene families using TreeFam [45]. Firstly, we collected protein sequences longer than 33 amino acids from these eight species. The longest protein isoform was retained from each gene. Secondly, blastp was used for all the protein sequence alignments against itself with an E-value of 1E-7. After alignment, fragmental alignments for each gene pair were conjoined using Solar [46]. Thirdly, gene families were constructed. We used average distance for the hierarchical clustering algorithm, requiring the minimum edge weight (H-score) of 10 and the minimum edge density (total number of edges/theoretical number of edges) of larger than 1/3. We used genes from other species as a reference to remove the redundancies of protein. Firstly, we choose gene families with more than two genes, and carry out alignment at protein level. Then, we check the location of each sequence and remove any two (or more) sequences with no overlap using genes from other species as a reference. Finally, 25,965 proteins were remained. Single-copy ortholog genes were used to construct a phylogenetic tree using PhyML with default parameters [47].

To further eliminate the homologous genes of relative species, 520 unique families and 6,341 un-clustered genes were carried out according to the following filter: 1) removal of homologs against transcripts of newt using a BLASTN alignment with E-value of 1E-5, and 1,777 CDSs were filtered; 2) removal of homologs against Western clawed frog and Tibetan frog protein sets using a BLASTN alignment with E-value of 1E-5, and 6,301 proteins were filtered. The rest of 3,839 proteins were defined as CGS-specific genes.

Functional annotation and KEGG enrichment

The functional annotation were aligned to three databases, non-redundant protein database (Nr) in NCBI, Swiss-Prot and Kyoto Encyclopedia of Genes and Genomes (KEGG) pathway database, by BLASTX (E-value ≤ 10^{-5}). Gene ontology (GO) classification was analyzed by the Blast2GO software (v2.5.0) based on Nr annotation.

KEGG pathway enrichment analysis for specific gene set was conducted based on an algorithm presented by KOBAS [48], using the entire coding gene set as the background. The *P*-value was approximated by a hypergeometric distribution test and multiple testing correction method. The cutoff of enriched pathways was a *P*-value of less than 0.05.

Estimation of gene expression and construction of co-expression network

For expression level, the clean reads of each sample were mapped to all transcripts using the Bowtie2 (version 2.2.5) software [49], then we used RSEM (v1.2.12) [50] to count the number of mapped reads and estimate FPKM (fragments per kilobase per million mapped fragments) values [51]. The tissue-specific expressed genes were detected by method based on Yu *et al* [52]. When a gene met two criteria of the expression enrichment (EE) score ≥ 2.5 and its *P*-value ≤ 0.001, it was defined as specifically expressed gene. Gene co-expression networks were constructed using WGCNA (Weighted gene co-expression network analysis) (Version: 1.48) [24]. A total of 26,135 coding genes as input were imported into WGCNA with the following settings: the power of 16, TOMType signed, minModuleSize of 30, and

mergeCutHeight of 0.25. The eigengene value was calculated for each module and used to test the association between each tissue. Module membership (known as module eigengene based connectivity kME) values were referred to as intramodular hub genes. The hub gene was statistically analyzed by WGCAN using cutoff of *P*-value < 1e-8 and kME > 0.95 in each module.

Availability of supporting data

The datasets supporting the results of this article are included within the article and its additional files. All the clean reads were deposited in the National Center for Biotechnology Information (NCBI) and could be accessed in the Short Read Archive (SRA accession: SRP092015) linking to BioProject accession number PRJNA350354. The assemblies and annotations data and other relevant data have also been hosted in the GigaScience GigaDB repository

Declarations

Acknowledgements

This work was supported by grants from the National Natural Science Foundation of China (No. 31572270), and the Major Scientific and Technological Projects of Henan (No. 111100910600). The funders had no role in study design, data collection and analysis, decision to publish, or preparation of the manuscript.

Competing interests

The authors declare that they have no competing interests.

Authors’ contributions

CSX, XDF and HW conceived the study and designed the experiments. HTS, QG,

XYZ and JLG performed the experiments. WSL, XFG, JL and JBJ analyzed the data. BHZ, YW, BC, ZGQ, MHZ, FCZ, JM and JBJ contributed reagents/materials/analysis tools. XFG and WSL wrote the manuscript with input from all authors. CSX, HW, FCZ and JM revised the paper. All authors read and approved the final manuscript.

Additional files

Additional file 1: Summary statistics of sequencing data and Q20 percentage.

Additional file 2: Hierarchical clustering of gene expression profiling. Coding genes (left); non-coding genes (right). The coding genes have higher expression abundances than non-coding genes.

Additional file 3: The expression profiling of all genes.

Additional file 4: Gene families among *A. davidianus*, *X. tropicalis*, *N. parkeri* and *P. sinensis*.

Additional file 5: KEGG pathway anlysis of CGS-specific genes.

Additional file 6: CGS-specific genes, gene expansion and pathways related to longevity and starvation tolerance.

Additional file 7: Sample clustering by WGCAN.

Additional file 8: Tissue-specific expressed genes and secreted proteins among three different parts of skin tissue. (A) Tissue-specific expressed gene. (B) Secreted proteins.

Additional file 9: Expression profilling of genes in the black module.

Additional file 10: The list of secreted proteins.

Additional file 11: The list of antibacterial peptides.

Reference

1. Zhao E, Hu Q, Jiang Y, Yang Y. Studies on Chinese salamanders. Society for the study of amphibians and reptiles Oxford, Ohio, U.S.A.1998.
2. Gao KQ, Shubin NH. Earliest known crown-group salamanders. Nature. 2003;422:424-8.
3. Zhu R, Chen ZY, Wang J, Yuan JD, Liao XY, Gui JF, et al. Extensive diversification of MHC in Chinese giant salamanders Andrias davidianus (Anda-MHC) reveals novel splice variants. Dev Comp Immunol. 2014;42:311-22.
4. Zhu R, Chen ZY, Wang J, Yuan JD, Liao XY, Gui JF, et al. Thymus cDNA library survey uncovers novel features of immune molecules in Chinese giant salamander Andrias davidianus. Dev Comp Immunol. 2014;46:413-22.
5. Zhu B, Feng Z, Qu A, Gao H, Zhang Y, Sun D, et al. Brief report. The karyotype of the caudate amphibian Andrias davidianus. Hereditas. 2002;136:85-8.
6. Jorgensen CB. Amphibian respiration and olfaction and their relationships: from Robert Townson (1794) to the present. Biol Rev Camb Philos Soc. 2000;75:297-345.
7. Xu X, Lai R. The chemistry and biological activities of peptides from amphibian skin secretions. Chem Rev. 2015;115:1760-846.
8. Fan Y, Chang MX, Ma J, LaPatra SE, Hu YW, Huang L, et al. Transcriptomic analysis of the host response to an iridovirus infection in Chinese giant salamander, Andrias davidianus. Vet Res. 2015;46:136.
9. Li F, Wang L, Lan Q, Yang H, Li Y, Liu X, et al. RNA-Seq analysis and gene discovery of Andrias davidianus using Illumina short read sequencing. PLoS One. 2015;10:e0123730.
10. Qi Z, Zhang Q, Wang Z, Ma T, Zhou J, Holland JW, et al. Transcriptome analysis of the endangered Chinese giant salamander (Andrias davidianus): Immune modulation in response to Aeromonas hydrophila infection. Vet Immunol Immunopathol. 2016;169:85-95.
11. Grabherr MG, Haas BJ, Yassour M, Levin JZ, Thompson DA, Amit I, et al. Full-length transcriptome assembly from RNA-Seq data without a reference genome. Nat Biotechnol. 2011;29:644-52.
12. Simao FA, Waterhouse RM, Ioannidis P, Kriventseva EV, Zdobnov EM. BUSCO: assessing genome assembly and annotation completeness with single-copy orthologs. Bioinformatics. 2015;31:3210-2.
13. Ziv E, Hu D. Genetic variation in insulin/IGF-1 signaling pathways and longevity. Ageing Res Rev. 2011;10:201-4.
14. Bartke A. Impact of reduced insulin-like growth factor-1/insulin signaling on aging in mammals: novel findings. Aging Cell. 2008;7:285-90.
15. Masse I, Molin L, Billaud M, Solari F. Lifespan and dauer regulation by tissue-specific activities of Caenorhabditis elegans DAF-18. Dev Biol. 2005;286:91-101.
16. Longo VD. The Ras and Sch9 pathways regulate stress resistance and longevity. Exp Gerontol. 2003;38:807-11.
17. Slack C, Alic N, Foley A, Cabecinha M, Hoddinott MP, Partridge L. The Ras-Erk-ETS-Signaling Pathway Is a Drug Target for Longevity. Cell. 2015;162:72-83.
18. Hwang AB, Ryu EA, Artan M, Chang HW, Kabir MH, Nam HJ, et al. Feedback regulation via AMPK and HIF-1 mediates ROS-dependent longevity in Caenorhabditis elegans. Proc Natl Acad Sci U S A. 2014;111:E4458-67.

19. Pickering AM, Lehr M, Miller RA. Lifespan of mice and primates correlates with immunoproteasome expression. J Clin Invest. 2015;125:2059-68.

20. Heimbucher T, Hunter T. The C. elegans Ortholog of USP7 controls DAF-16 stability in Insulin/IGF-1-like signaling. Worm. 2015;4:e1103429.

21. Fruhbeck G, Mendez-Gimenez L, Fernandez-Formoso JA, Fernandez S, Rodriguez A. Regulation of adipocyte lipolysis. Nutr Res Rev. 2014;27:63-93.

22. Ozcan L, Wong CC, Li G, Xu T, Pajvani U, Park SK, et al. Calcium signaling through CaMKII regulates hepatic glucose production in fasting and obesity. Cell Metab. 2012;15:739-51.

23. Hall JA, Tabata M, Rodgers JT, Puigserver P. USP7 attenuates hepatic gluconeogenesis through modulation of FoxO1 gene promoter occupancy. Mol Endocrinol. 2014;28:912-24.

24. Langfelder P, Horvath S. WGCNA: an R package for weighted correlation network analysis. BMC Bioinformatics. 2008;9:559.

25. Geng X, Wei H, Shang H, Zhou M, Chen B, Zhang F, et al. Proteomic analysis of the skin of Chinese giant salamander (Andrias davidianus). J Proteomics. 2015;119:196-208.

26. Sun J, Geng X, Guo J, Zang X, Li P, Li D, et al. Proteomic analysis of the skin from Chinese fire-bellied newt and comparison to Chinese giant salamander. Comp Biochem Physiol Part D Genomics Proteomics. 2016;19:71-7.

27. Amagai M, Stanley JR. Desmoglein as a target in skin disease and beyond. J Invest Dermatol. 2012;132:776-84.

28. Shibata Y, Sano T, Tsuchiya N, Okada R, Mochida H, Tanaka S, et al. Gene expression and localization of two types of AQP5 in Xenopus tropicalis under hydration and dehydration. Am J Physiol Regul Integr Comp Physiol. 2014;307:R44-56.

29. Willumsen NJ, Amstrup J, Mobjerg N, Jespersen A, Kristensen P, Larsen EH. Mitochondria-rich cells as experimental model in studies of epithelial chloride channels. Biochim Biophys Acta. 2002;1566:28-43.

30. Lazado CC, Lund I, Pedersen PB, Nguyen HQ. Humoral and mucosal defense molecules rhythmically oscillate during a light-dark cycle in permit, Trachinotus falcatus. Fish Shellfish Immunol. 2015;47:902-12.

31. Perez-Sanchez J, Estensoro I, Redondo MJ, Calduch-Giner JA, Kaushik S, Sitja-Bobadilla A. Mucins as diagnostic and prognostic biomarkers in a fish-parasite model: transcriptional and functional analysis. PLoS One. 2013;8:e65457.

32. Slominski A, Zbytek B, Nikolakis G, Manna PR, Skobowiat C, Zmijewski M, et al. Steroidogenesis in the skin: implications for local immune functions. J Steroid Biochem Mol Biol. 2013;137:107-23.

33. Itoi-Ochi S, Terao M, Murota H, Katayama I. Local corticosterone activation by 11beta-hydroxysteroid dehydrogenase 1 in keratinocytes: the role in narrow-band UVB-induced dermatitis. Dermatoendocrinol. 2016;8:e1119958.

34. Grall A, Guaguere E, Planchais S, Grond S, Bourrat E, Hausser I, et al. PNPLA1 mutations cause autosomal recessive congenital ichthyosis in golden retriever dogs and humans. Nat Genet. 2012;44:140-7.

35. Chen T, Farragher S, Bjourson AJ, Orr DF, Rao P, Shaw C. Granular gland transcriptomes in stimulated amphibian skin secretions. Biochem J. 2003;371:125-30.

36. Missan DS, Chittur SV, DiPersio CM. Regulation of fibulin-2 gene expression by integrin alpha3beta1 contributes to the invasive phenotype of transformed keratinocytes. J Invest

Dermatol. 2014;134:2418-27.

37. Ye X, Wang Y, Cahill H, Yu M, Badea TC, Smallwood PM, et al. Norrin, frizzled-4, and Lrp5 signaling in endothelial cells controls a genetic program for retinal vascularization. Cell. 2009;139:285-98.

38. Mulero JJ, Boyle BJ, Bradley S, Bright JM, Nelken ST, Ho TT, et al. Three new human members of the lipid transfer/lipopolysaccharide binding protein family (LT/LBP). Immunogenetics. 2002;54:293-300.

39. Krane IM, Naylor SL, Helin-Davis D, Chin WW, Spindel ER. Molecular cloning of cDNAs encoding the human bombesin-like peptide neuromedin B. Chromosomal localization and comparison to cDNAs encoding its amphibian homolog ranatensin. J Biol Chem. 1988;263:13317-23.

40. Itoh S, Takashima A, Itoh T, Morimoto T. Open-field behavior of rats following intracerebroventricular administration of neuromedin B, neuromedin C, and related amphibian peptides. Jpn J Physiol. 1994;44:271-81.

41. Shao H, Cai L, Grichnik JM, Livingstone AS, Velazquez OC, Liu ZJ. Activation of Notch1 signaling in stromal fibroblasts inhibits melanoma growth by upregulating WISP-1. Oncogene. 2011;30:4316-26.

42. Lai A, Ghaffari A, Li Y, Ghahary A. Paracrine regulation of fibroblast aminopeptidase N/CD13 expression by keratinocyte-releasable stratifin. J Cell Physiol. 2011;226:3114-20.

43. Sun C, Shepard DB, Chong RA, Lopez Arriaza J, Hall K, Castoe TA, et al. LTR retrotransposons contribute to genomic gigantism in plethodontid salamanders. Genome Biol Evol. 2012;4:168-83.

44. Sun C, Mueller RL. Hellbender genome sequences shed light on genomic expansion at the base of crown salamanders. Genome Biol Evol. 2014;6:1818-29.

45. Li H, Coghlan A, Ruan J, Coin LJ, Heriche JK, Osmotherly L, et al. TreeFam: a curated database of phylogenetic trees of animal gene families. Nucleic Acids Res. 2006;34:D572-80.

46. Yu XJ, Zheng HK, Wang J, Wang W, Su B. Detecting lineage-specific adaptive evolution of brain-expressed genes in human using rhesus macaque as outgroup. Genomics. 2006;88:745-51.

47. Guindon S, Dufayard JF, Lefort V, Anisimova M, Hordijk W, Gascuel O. New algorithms and methods to estimate maximum-likelihood phylogenies: assessing the performance of PhyML 3.0. Syst Biol. 2010;59:307-21.

48. Xie C, Mao X, Huang J, Ding Y, Wu J, Dong S, et al. KOBAS 2.0: a web server for annotation and identification of enriched pathways and diseases. Nucleic Acids Res. 2011;39:W316-22.

49. Langmead B, Salzberg SL. Fast gapped-read alignment with Bowtie 2. Nat Methods. 2012;9:357-9.

50. Li B, Dewey CN. RSEM: accurate transcript quantification from RNA-Seq data with or without a reference genome. BMC Bioinformatics. 2011;12:323.

51. Mortazavi A, Williams BA, McCue K, Schaeffer L, Wold B. Mapping and quantifying mammalian transcriptomes by RNA-Seq. Nat Methods. 2008;5:621-8.

52. Yu X, Lin J, Zack DJ, Qian J. Computational analysis of tissue-specific combinatorial gene regulation: predicting interaction between transcription factors in human tissues. Nucleic Acids Res. 2006;34:4925-36.

Tables

Table 1. The statistics of final assembly and coding gene prediction.

total data (Mb)	total length (bp)	total number (⩾250bp)	total number (⩾1kp)	average length	coding gene	non-coding genes
156,347	123,835,135	93,366	34,840	1,326	26,135	67,231

Table 2. The statistics of expressed transcripts, coding genes, secreted proteins and tissue-specific expressed sequences.

samples	expressed transcripts	coding genes	secreted proteins	tissue-specific expressed gene	tissue-specific expressed lncRNA
abdominal skin	53,324	20,193	1,584	139	253
dorsal skin	60,446	21,580	1,694	127	161
lateral skin	53,285	20,437	1,539	108	227
blood	56,540	19,994	1,387	223	474
brain	66,923	22,715	1,830	596	1,206
cartilage	59,724	20,979	1,684	282	603
eye	67,769	22,826	1,870	123	137
fat	65,586	21,570	1,632	70	375
fingertip	63,582	21,626	1,611	155	393
heart	62,127	21,734	1,691	253	459
kidney	66,223	22,792	1,821	381	467
liver	59,755	21,622	1,727	504	535
long bone	56,286	19,754	1,515	854	6,739
lung	70,132	22,991	1,820	108	147
maxillary	59,431	21,424	1,713	55	138
muscle	49,582	19,968	1,558	402	624
ovary	53,343	21,072	1,672	2,001	1,314
pancreas	44,177	18,746	1,546	276	416
skull	59,933	22,206	1,788	173	261
small intestine	59,156	21,588	1,721	608	730
spinal cord	64,808	22,423	1,801	499	1,029
spleen	64,258	21,699	1,685	225	327
stomach	58,688	21,601	1,759	193	292
tail fat	63,090	21,264	1,643	123	168

Table 3. The results of gene family classification.

species	total genes	un-clustered genes	gene families	unique families	average genes per family
A. davidianus	26,135(25,965)*	6,341	12,188	520	1.62
X.tropicalis	18,429	218	13,235	21	1.38
N.parkeri	22,972	2,391	13,986	306	1.47
A.carolinensis	17,767	818	13,387	30	1.27
P.sinensis	18,164	638	13,548	31	1.29
D.rerio	26,046	1,453	13,832	177	1.78
O.latipes	19,671	1,461	12,437	138	1.46
H.sapiens	21,375	2,062	15,542	409	1.24

Asterisk (*) represents gene number after correction.

Figure captions

Fig. 1. Huge RNA-seq data assembly. (A) The pipeline for de novo assembly, quality filter, and gene identification and classification. (B) The statistics of mapping rate before and after transcripts filter. Compared to total mapping ratio, the unique mapping rate was less than 2%, except sample 'stomach'. Moreover, the total mapping rate was a slight decrease in comparison to the result before filter.

Fig. 2. Identification and evaluation of giant salamander gene set. (A) The pipeline of prediction of coding genes. PRD represents Western clawed frog protein set, 947 proteins of CGS and 554 proteins of Newt from NCBI. (B) The results of BUSCO estimation. Asterisk (*) represents the final protein sets; pound ($^{\#}$) represents the primary protein sets. (C) Comparison of the length of homologous region to *X. tropicalis* and *N. parkeri*. The X-aixs is the ratio of length, and the Y-aixs is the percentage of gene number. (D) Comparison of the length of homologous sequence to *X. tropicalis* and *N. parkeri*. The X-aixs is log base 2 of length, and the Y-aixs is the percentage of gene number.

Fig. 3. CGS-specific genes, gene expansion and pathways related to longevity and starvation tolerance. The number in bracket represents the number of CGS-specific genes; gray background represents that the pathway is positive to longevity; protein in red font represents that this protein has more gene copies than Western clawed frog and Tibetan frog; red line border represents the pathway significant expansion to Western clawed frog and Tibetan frog.

Fig. 4. Heatmap illuminates the association between modules and tissues. Each row corresponds to a module. Each column corresponds to a specific sample. The color of each cell at the row-column intersection indicates the correlation coefficient between the module and the sample. A high degree of correlation between a specific module and the sample is indicated by dark red.

Fig. 5. Co-expression network and expression profiling of each pathway using genes in the black module. The gene in network was not illumated when the weight is less than 0.2. The triangle represents hub genes, which is statistically analyzed by WGCAN using cutoff of P-value < 1e-8 and kME > 0.95 in each module. (A) Biosynthesis. (B) Melanin. (C) Immunity. (D) Metabolism. (E) Secretion. (F) Total expression profiling of each pathway using genes in black module.

Figure 1

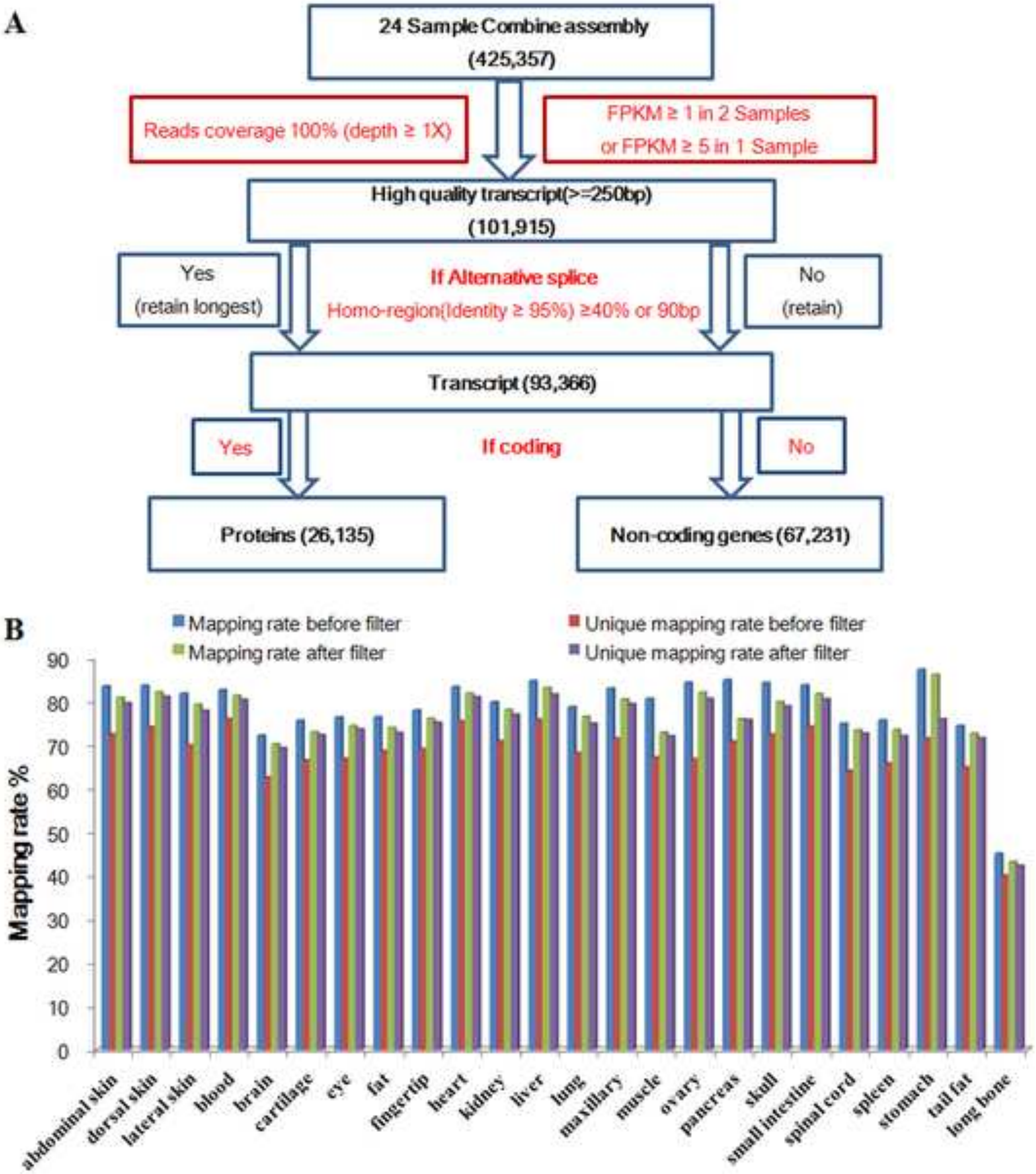
A
24 Sample Combine assembly
(425,357)
Reads coverage 100% (depth ≥ 1X)
FPKM ≥ 1 in 2 Samples
or FPKM ≥ 5 in 1 Sample
High quality transcript(>=250bp)
(101,915)
Yes
(retain longest)
If Alternative splice
Homo-region(Identity ≥ 95%) ≥40% or 90bp
No
(retain)
Transcript (93,366)
Yes
If coding
No
Proteins (26,135)
Non-coding genes (67,231)
B
Mapping rate before filter
Mapping rate after filter
Unique mapping rate before filter
Unique mapping rate after filter
Mapping rate %
90
80
70
60
50
40
30
20
10
0
abdominal skin
dorsal skin
lateral skin
blood
brain
cartilage
eye
fat
fingertip
heart
kidney
liver
lung
maxillary
muscle
ovary
pancreas
skull
small intestine
spinal cord
spleen
stomach
tail fat
long bone

Figure 2

Click here to download Figure Figure 2.tif

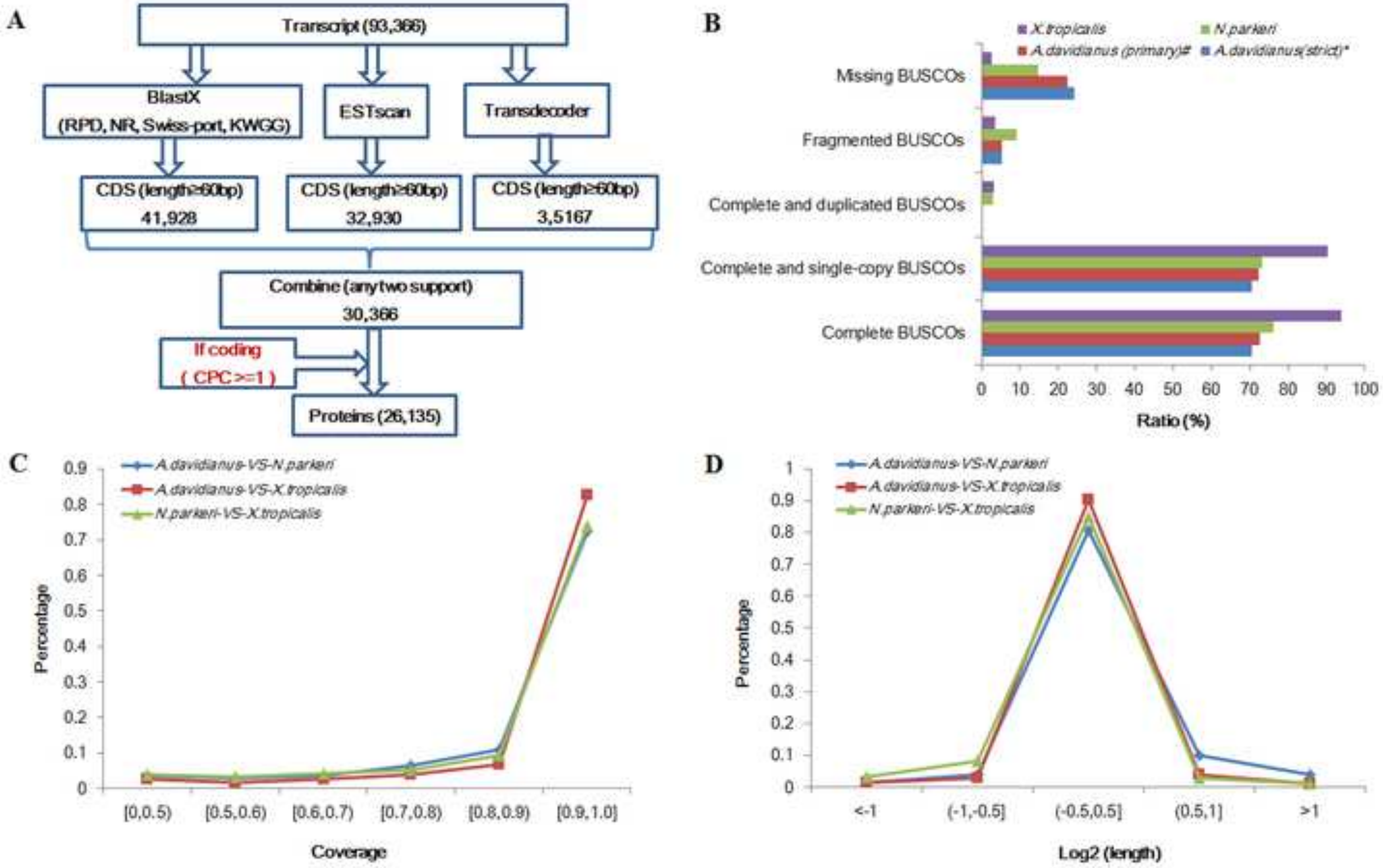

Figure 3 Click here to download Figure Figure 3.tif

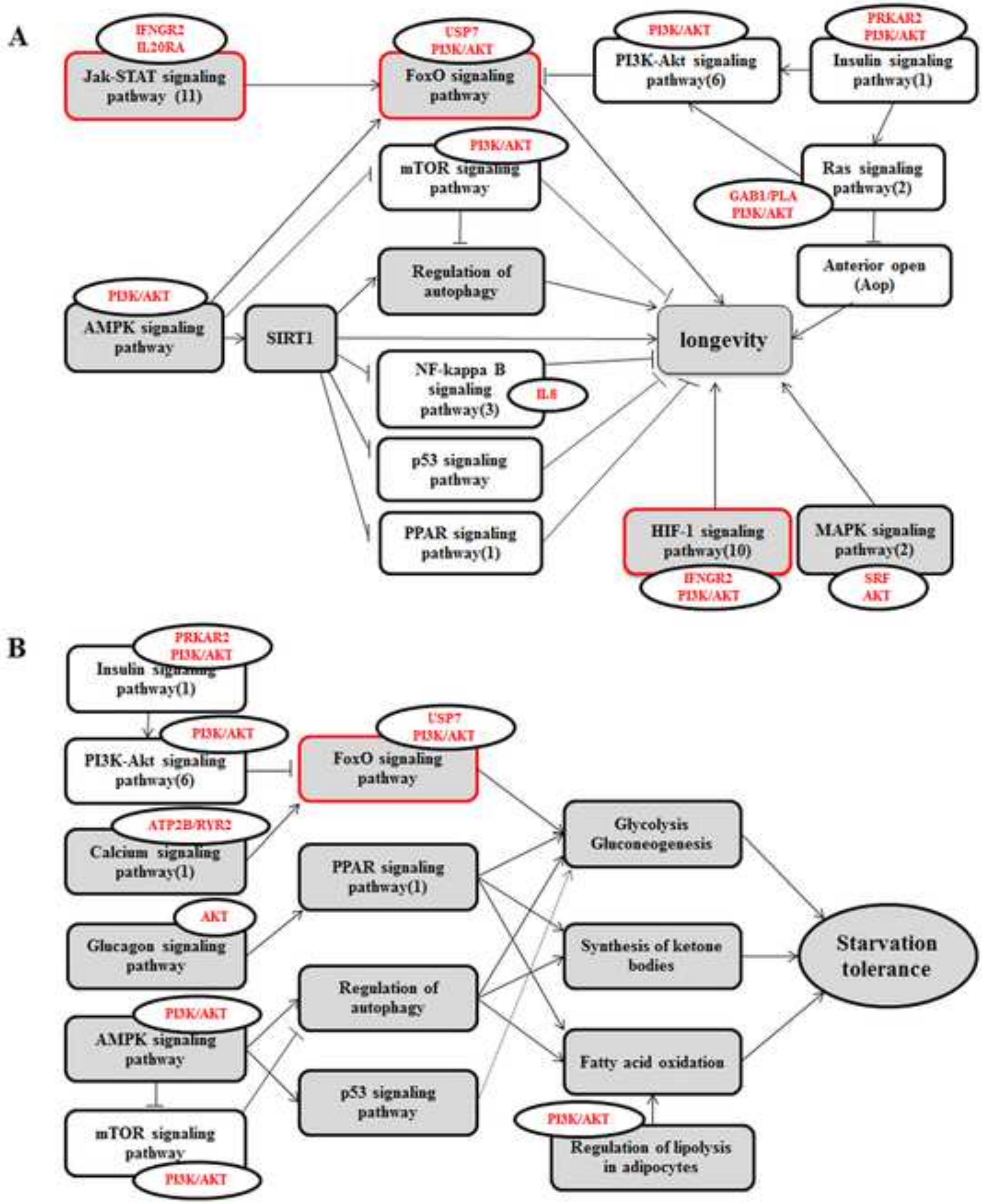

Figure 4

Click here to download Figure Figure 4.tif

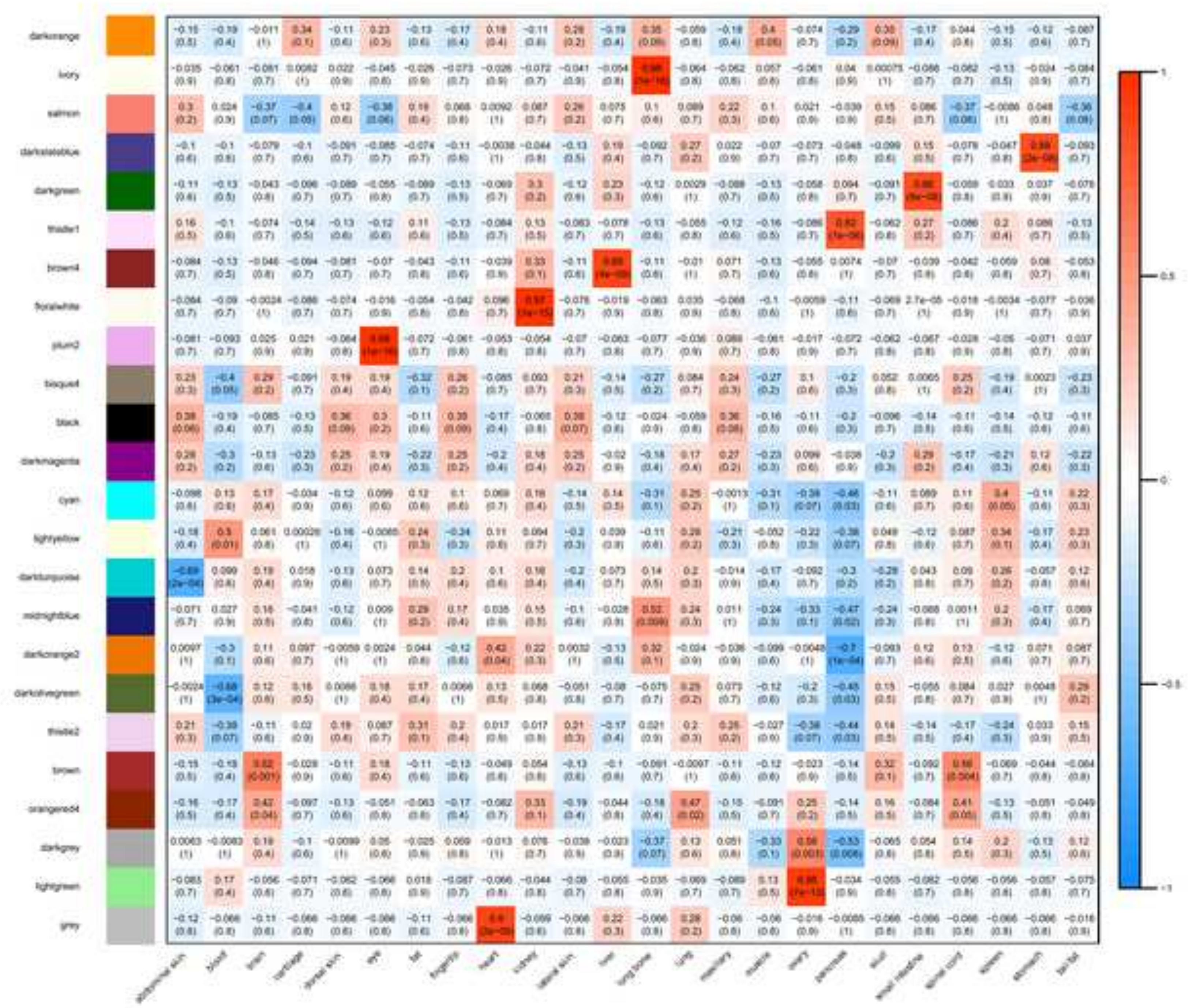

Figure 5 Click here to download Figure Figure 5.tif

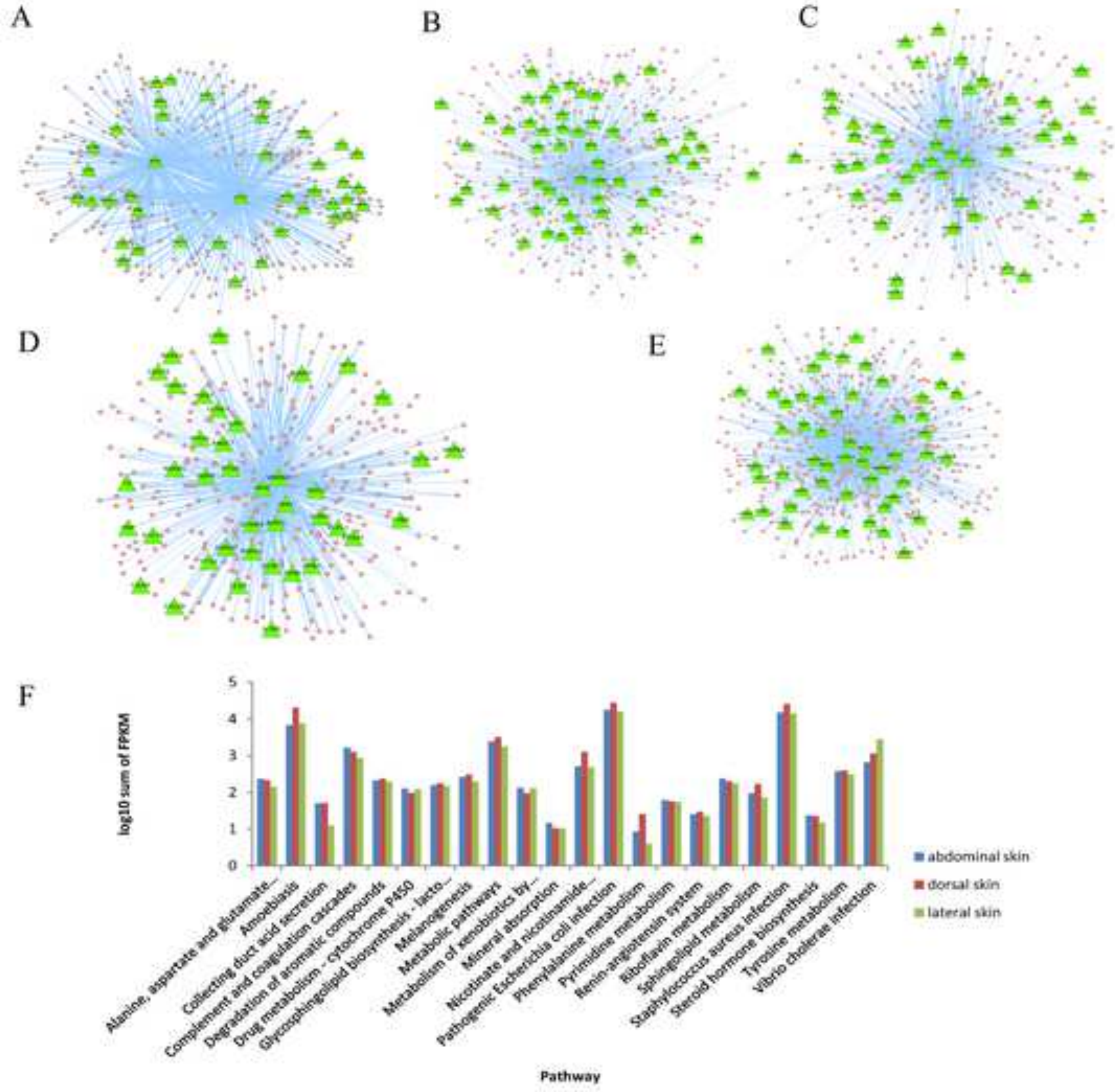

Click here to access/download
Supplementary Material
Additional file 1.docx

Click here to access/download
Supplementary Material
Additional file 2.xlsx

Click here to access/download
Supplementary Material
Additional file 3.tif

Click here to access/download
Supplementary Material
Additional file 4.tif

Click here to access/download
Supplementary Material
Additional file 5.xlsx

Click here to access/download
Supplementary Material
Additional file 6.xlsx

Click here to access/download
Supplementary Material
Additional file 7.tif

Click here to access/download
Supplementary Material
Additional file 8.tif

Click here to access/download
Supplementary Material
Additional file 9.xlsx

Click here to access/download
Supplementary Material
Additional file 10.xlsx

Click here to access/download
Supplementary Material
Additional file 11.xlsx

1. The raw data needs to be made available in the SRA, as it currently cannot be viewed. The SUB number you quote is just the submission number you get automatically from NCBI when they start a submission, and is not an actual submission. We would need the actual BioProject accession (PRJ……), and then switch it live or provide reviewer access if its not public yet (NCBI can provide this).

Answer: Thank you for your suggestion. All the clean reads were deposited in the National Center for Biotechnology Information (NCBI) and could be accessed in the Short Read Archive database (accession number: SRP092015) linking to BioProject accession number: PRJNA350354.

Reviewer can link to metadata by the following website:

ftp://ftp-trace.ncbi.nlm.nih.gov/sra/review/SRP092015_20161102_135359_27e795eb0f314edf0479737480ab0f2a

2. Please provide the assemblies and annotations for us, and any other relevant custom scripts or analysis results relevant for reproducibility. I have cc'd our curators and will provide FTP access for you to copy this over.

Answer: Thank you for your suggestion. The assemblies and annotations data and other relevant data have been hosted in the GigaScience GigaDB repository by FTP. The data are listed as folders under the file "GigaScience, GIGA-D-16-00117".

3. Is any of this data in common with the BGI Chinese Giant Salamander project, as it would be good to collaborate and credit with the genome sequencing efforts. There are 2 other public SRA studies of transcriptome data from the same species, it would have been nice for you to include/compare that data for this. Even without this, please cite data accessions for the two frog genomes you compare to. And we will like to see the Sample accessions for each of the different tissues too, but if its accepted can get these from you then.

Answer: Thank you for your suggestion. Although the transcriptome data of skin and spleen (SRA accession number: SRP048762) from Chinese Giant Salamander have been deposited in NCBI, this study only sequenced two different tissues. Therefore, these data cannot compare with our data of 24 samples. The transcriptome data of Western clawed frog (*Xenopus tropicalis*) was obtained from website (http://ftp.ensembl.org/pub/release-81/fasta/ xenopus_tropicalis/), and the transcriptome data of Tibetan frog (*Nanorana parkeri*) was deposited in NCBI with BioProject

accession PRJNA243398.

4. CEGMA is outdated so please provide BUSCO results with or instead of this.

Answer: Thank you for your suggestion. We have deleted CEGMA method and employed BUSCO (Benchmarking Universal Single-Copy Orthologs; http://busco.ezlab.org/) method to estimate the completeness of this coding gene set on CGS (*Andrias davidianus*), and compared it with the two frog species Western clawed frog (*Xenopus tropicalis*) and Tibetan frog (*Nanorana parkeri*). The total number of genes for evaluation is 3023. Nearly 70.6 % of total complete and single-copy BUSCOs were identified in this gene set and 73.3 % (Tibetan frog) and 90.4% (Western clawed frog) of this indictor in two frogs' gene sets (Fig. 2B). The 'Complete and duplicated BUSCOs' nearly zero in CGS compare to 2.8% and 3.4% in two frogs (Fig. 2B). This data showed that our gene set had low duplicates. And the ratio of 'Fragmented BUSCOs' is 5.2%, more than Western clawed frog (3.6%) and less than Tibetan frog (9.1%) (Fig. 2B). These data hinted that we obtained a good gene sets by RNA-seq with quality metrics comparable to gene set of Tibetan frog.

5. If the reviewers think there isn't enough wetlab validation you might need to add that (getting hold of the proteomics data might help), or we can change it to a Data Note article. We will see what the reviewers think regarding this point, but we thought we would give you a warning about this in advance.

Answer: Thanks for your advice. If the manuscript cannot be published in research article, we agree that you change it to a Data Note article.

Sincerely yours,

Dr. Xiaofang Geng

Corresponding authors: Prof. Cunshuan Xu (cellkeylab@126.com); Prof. Xiaodong Fang (fangxd@genomics.cn); Prof. Hong Wei (weihong63528@163.com)
