# Supplementary material for: A reference gene set construction using RNA-seq of multiple tissues of Chinese giant salamander, Andrias davidianus
Source: Gigascience. 2017 Feb 15;6(3):1–7. doi: 10.1093/gigascience/gix006 (PMC5467019; doi:10.1093/gigascience/gix006)
Supplement: GIGA-D-16-00117_Revision_1.pdf [file gix006_giga-d-16-00117_revision_1.pdf]

Manuscript

Click here to download Manuscript Manuscript-DataNote v2.docx

A reference gene set construction using RNA-seq of multiple tissues of Chinese Giant Salamander, *Andrias davidianus*

Xiaofang Geng[1,5]¶, Wanshun Li[3]¶, Haitao Shang[2]¶, Qiang Gou[4], Fuchun Zhang[5], Xiayan Zang[1], Benhua Zeng[2], Jiang Li[3], Ying Wang[4], Ji Ma[5], Jianlin Guo[1], Jianbo Jian[3], Bing Chen[4], Zhigang Qiao[1], Minghui Zhou[4], Hong Wei[2]*, Xiaodong Fang[3]*, Cunshuan Xu[1]*

[1] State Key Laboratory Cultivation Base for Cell Differentiation Regulation, College of Life Science, Henan Normal University, Xinxiang, China

[2] Department of Laboratory Animal Science, College of Basic Medical Sciences, Third Military Medical University, Chongqing, China

[3] BGI-Shenzhen, Shenzhen, China

[4] Chongqing Kui Xu Biotechnology Incorporated Company, Kaixian Country, Chongqing, China

[5] Xinjiang Key Laboratory of Biological Resources and Genetic Engineering, College of Life Science and Technology, Xinjiang University, Urumqi, China

* Corresponding author

E-mail: cellkeylab@126.com (CSX); fangxd@genomics.cn (XDF); weihong63528@163.com (HW)

¶These authors contributed equally to this work.

Abstract

Background: Chinese giant salamander (CGS) is the largest extant amphibian species in the world. Owing to its evolutionary position and four peculiar phenomenon of life (longevity, starvation tolerance, regenerative ability, and hatch without sunshine), it is an invaluable model species for research. However, lack of genomic resources leads to fewer study progresses in these field, due to its huge genome of ~50 GB extremely difficult to be assembled.

Results: We reported the sequenced transcriptome of more than twenty tissues from adulthood of CGS using Illumina Hiseq 2000 technology, and a total of 93,366 no redundancy transcripts with a mean length of 1,326 bp were obtained. We for the first time developed an efficient pipeline to construct a high quality reference gene set of CGS and obtained 26,135 coding genes. BUSCO and homologous assessment showed that our assembly captured 70.6 % of vertebrate universal single-copy orthologs, and this coding gene set had a higher proportion of completeness CDS with comparable quality of the protein sets of Tibetan frog.

Conclusions: These highest quality data will provide valuable reference gene set to the subsequent research of CGS. In addition, our strategy of de novo transcriptome assembly and protein identification is applicable to similar studies.

Keywords: Chinese Giant Salamander; *Andrias davidianus*; *De novo* transcriptome; Assembly

Data description

Background

The Chinese giant salamander (CGS; Andrias davidianus), belonging to order Caudata, family Cryptobranchidae, is the largest extant amphibian species in the world. It is endemic to mainland China and widely distributed in central, south-western and southern China. It is crowned as a living fossil because it has existed for more than 350 million years [1]. It is an invaluable model species for research in the fields of evolution and phylogeny, owing to its important evolutionary position representing the transition of animal from aquatic to terrestrial life [2, 3]. However, in the past 50 years, the natural populations of CGS have sharply declined due to habitat destruction, climate change and overhunting. This endangered amphibian has now been listed in annex I of the Convention on International Trade in Endangered Species of Wild Fauna and Flora (CITES) and in class II of the national list of protected animals in China [4]. It has also been listed as one of the top 10 "focal species" in 2008 by the Evolutionarily Distinct and Globally Endangered (EDGE) project. Natural population decline and high values for scientific conservation and medicinal use lead to its commercial aquaculture in many locations throughout China.

Despite their unique life-history characteristics, this species remains poorly characterized at the molecular level. No genomic resources are available for this species, because it has larger genomes with about 50 GB and is extremely difficult to conduct whole-genome de novo assembly even with present sequencing technology. Fortunately, RNA sequencing technologies provide cost-effective alternative

approaches for the construction of the transcribed gene. Transcriptome analysis using Illumina sequencing technology has been reported in the skin and spleen of CGS [5-7], but these studies mainly discover genes associated with the immune and inflammatory response, and only two different tissues can't obtain enough genes to research specific biology of CGS. Here, we reported the sequenced transcriptome of more than twenty tissues from adult CGS using Illumina Hiseq 2000 technology. Our results showed that a reference gene set with high quality was constructed in this study, and it will serve as a valuable resource for future biology study of CGS.

Samples collection

Adult female Chinese giant salamanders with weight of about 2 kg were obtained from an artificial breeding base Chongqing Kui Xu Biotechnology Incorporated Company. The giant salamanders were reared in aerated, tap water supplied tanks at 20 °C and fed with diced bighead carp for 2 weeks prior to experiment. Animals were heavily anesthetized by anaesthetic MS-222 and sacrificed by dissection before sample collection. Multiple tissues (abdominal skin, dorsal skin, lateral skin, lung, heart, kidney, liver, pancreas, small intestine, spleen, stomach, brain, spinal cord, cartilage, eye, fingertip, long bone, maxillary, skull, muscle, ovary, fat, tail fat, blood) were collected.

Sequencing

Total RNA (~10 μg) was extracted from each sample using the Trizol Reagent (Invitrogen). The cDNA library was contracted using TruSeq®RNA sample prep kit (Illumina) according to its manufacturer's protocol. After quality control of the cDNA

libraries, pair-end sequencing was carried out *via* Illumina HiSeq™ 2000 at the Beijing Genomics Institute in Shenzhen. To ensure the accuracy of de novo assembly, raw reads were filtered by removal of adaptor and low quality sequences. After the preprocessing of reads, up to 156 GB of clean data were obtained in total, at least 6.4 GB of data in each sample with Q20 bases more than 96 % (Table 1).

Huge RNA-seq data assembly and evaluation

To obtain an integrated transcript set, we first put together all clean data and performed a combined assembly strategy by a publicly available program Trinity (V2.0.6; http://trinityrnaseq.sourceforge.net/) with the following parameters: min_kmer_cov=3, min_glue=3, group_pairs_distance=250, path_reinforcement_distance=85 [8]. It yields a huge number of transcripts, up to 425,357 transcripts output, and it includes lots of assembly errors and background sequences. To reduce these background and assembly errors, we developed a strict pipeline to filter these sequences (Fig. 1A). 1) Removal of assembly errors. Only each base pair in any sequence covered by at least one read will be saved, except 50 bp near to both end of sequence. Any gap will be trimmed, no matter where it is. The sequence will be split into pieces at gap sites, if there have gaps in the middle of sequence. 2) Removal of the background sequences. The clean reads were mapped to all the transcripts and FPKM value was calculated. When the expression profiling of sequence reached this standard of ≥ 1 FPKM in at least two samples or ≥ 5 FPKM in at least one sample, it will be retained. 3) Removal of isoforms produced by alternative splice. The high homologous region (identity at least 95 %) between two

sequences reaches to this criterion: larger than 40 % or 90 bp in length of one sequence, and the shorter one will be removed. 4) Removal of short sequences. The sequence with less than 250 bp in length will be discarded. Finally, a total of 93,366 transcripts with a mean length of 1,326 bp were obtained (Table 2). The clean reads were mapped to all the transcripts, the total mapping rate and unique mapping rate ranged from 70.15-86.07 % and 69.24-81.56 %, respectively, except sample 'long bone' (43.12 % and 42.21 %; Fig. 1B).

To evaluate our filtering pipeline, we compared the total mapping rate and unique mapping rate of transcripts before and after filter, respectively. After filter, the total mapping rate was a slight decrease (less than 2.6% in average) in comparison to before filter (Fig. 1B), and it suggested that we retained a higher completeness rate. On other hand, among the total mapped reads, the multiple mapped reads is less than 2 % in the transcripts after filter except sample 'stomach', while the same feather is up to 10.39 % (average ratio in 24 samples) in the transcripts before filter. This data hinted that most redundancy was removed and the set of transcripts after filter had very low redundancy. Above all, our filter pipeline made a good effective, not only removing the assembly error and redundancy, but also keeping most of the unique expressed sequences.

Functional annotation

A total of 41,874 sequences can be annotated by searching against four function databases, non-redundant protein database (Nr) in NCBI, Swiss-Prot and Kyoto Encyclopedia of Genes and Genomes (KEGG) pathway database, COG using

BLASTX (E-value ≤ 10^{-5}) (Table 3). Gene ontology (GO) classification was analyzed by the Blast2GO software (v2.5.0) based on Nr annotation.

Identification of coding gene set

To identify high-quality coding proteins, we developed the following pipeline to perform (Fig. 2A). Firstly, we predicted the CDS (coding sequences) of at least 60 bp using the following three methods. 1) We predicted the CDS using transdecoder (https://transdecoder.github.io/ version 2.0.1). 2) All transcripts were searched in protein databases using blastx (E-value < 10^{-5}) in the following order: PRD [western clawed frog protein set, 947 proteins of CGS and 554 proteins of newt from NCBI], Nr, SwissProt and KEGG. Transcripts with sequences having matches in one database were not searched further. We selected CDS from sequences based on the best hit. 3) All transcripts were used to predict the CDS by ESTScan (http://www.ch.embnet.org/software/ESTScan2.html; v3.0.2). Before prediction, the ESTScan was trained using the CDS data produced by blastx alignment method. The transcripts with CDS regions were identified by any two methods mentioned above and the longest CDS will be chosen. Then, we filtered them with these criteria: the shortest CDS was at least 100 bp and the ratio of cds/mRNA in length was at least more than 0.1. This data were defined as ‘primary protein sets’, and it will be checked in next step. Secondly, the candidate transcripts will be predicted by CPC (Coding Potential Calculator) software (http://cpc.cbi.pku.edu.cn/). When the transcript was reported as a coding gene and the score was no less than 1, it was defined as a true coding gene. At last, 26,135 sequences (25,965 genes after removing redundancy)

were passed our criterion, which were defined as coding genes, and the rest of 67,231 sequences were defined as non-coding genes (Table 2).

Evaluation of coding gene set

To evaluate the completeness of this coding gene set, we employed BUSCO (Benchmarking Universal Single-Copy Orthologs; http://busco.ezlab.org/) to evaluate the gene set of CGS using vertebrata data [9] and compared with two frog species, which have whole genome data available as follows, Western clawed frog (*Xenopus tropicalis*; http://ftp.ensembl.org/pub/release-81/fasta/xenopus_tropicalis/) and Tibetan frog (*Nanorana parkeri*; BioProject accession: PRJNA243398). The total number of genes for evaluation is 3023. Nearly 70.6 % of total complete and single-copy BUSCOs were identified in this gene set and 73.3 % (Tibetan frog) and 90.4% (Western clawed frog) of this indictor in two frogs' gene sets (Fig. 2B). The 'Complete and duplicated BUSCOs' nearly zero in CGS compare to 2.8% and 3.4% in two frogs (Fig. 2B). This data showed that our gene set had low duplicates. And the ratio of 'Fragmented BUSCOs' is 5.2%, more than Western clawed frog (3.6%) and less than Tibetan frog (9.1%) (Fig. 2B). These data hinted that we obtained an acceptable gene set of CGS which has comparable quality of whole genome sequencing of Tibetan frog, although we only used dozens of sample by RNA-seq. We also performed the same analysis using the primary protein sets, which were only identified by three kinds of CDS prediction methods (see Methods). Fortunately, these two results were much closed (70.6 % and 72.3 %) (Fig. 2B). These data suggest that CPC (Coding Potential Calculator) method has highly effective to remove non-coding

RNAs and remain the coding mRNAs.

We advanced to evaluate the completeness of single gene using single copy genes among three amphibian species. To identify the single copy gene families, we selected the following reference species: *A. davidianus*, *X. tropicalis*, *N. parkeri*, *A. carolinensis*, *P. sinensis*, *D. rerio*, *O. latipes* and *H. sapiens*. For comparative analysis, we used the following pipeline to cluster individual genes into gene families using TreeFam [10]. Firstly, we collected protein sequences longer than 33 amino acids from these eight species. The longest protein isoform was retained from each gene. Secondly, blastp was used for all the protein sequence alignments against itself with an E-value of 1E-7. After alignment, fragmental alignments for each gene pair were conjoined using Solar [11]. Thirdly, gene families were constructed. We used average distance for the hierarchical clustering algorithm, requiring the minimum edge weight (H-score) of 10 and the minimum edge density (total number of edges/theoretical number of edges) of larger than 1/3. The result of family classification showed that CGS obtained slight less gene families than two flogs (Table 4). Finally, we identified 6,634 single copy genes among three amphibian species mentioned above to evaluate the completeness of single CDS. The results showed that the percentage of CGS's CDS with at least 90 % homologous regions in Western clawed frog's ortholog (more than 82 %) was higher than Tibetan frog-vs-Western clawed frog (74%; Fig. 2C) and CGS-vs-Tibetan frog (73%; Fig. 2C). Moreover, the CDS length of CGS was closer to Western clawed frog than Tibetan frog, and they had longer CDS than Tibetan frog (Fig. 2D). Considering differences among species, this data showed that we have

obtained a higher proportion of completeness CDS in this gene set.

Estimation of gene expression

For expression level, the clean reads of each sample were mapped to all transcripts using the Bowtie2 (version 2.2.5) software [12], then we used RSEM (v1.2.12) [13] to count the number of mapped reads and estimate FPKM (fragments per kilobase per million mapped fragments) values [14]. The expressed transcripts ranged from 47.32 % to 75.12 % of 93,366 total transcripts in each library. The expressed transcripts and genes number was illuminated in Table 5 and detail profiling of all genes was summarized in Additional file 1. The hierarchical clustering of gene expression profiling was analyzed, and the results showed that the coding genes had higher expression level than non-coding genes (Fig. 3).

Conclusions and future directions

In summary, we sequenced 24 RNA-seq samples from adult of CGS to construct a good reference gene set in this study, due to CGS with a huge genome size of ~50 GB, which was hardly constructed well by present sequencing technology. A total of 26,135 coding genes with comparable quality of protein sets of Tibetan frog were identified; CGS has more gene number than Western clawed frog of 18,429 proteins and Tibetan frog of 22,972 proteins. Moreover, this coding gene set contains approximately 70 % of Universal Single-Copy Orthologs of vertebrata genes, and had a higher proportion of completeness CDS with quality metrics comparable to gene set of Tibetan frog. Gene families obtained in CGS were slightly less than the other two amphibian species. Hence, we believe that CGS has more gene number than Western

clawed frog and Tibetan frog. The most likely is that more gene copies were produced with transposon element expansion and low loss rate. Sun et al. [15, 16] reported that LTR retrotransposons expansion contributed to genomic gigantism of several salamanders. Similar mechanism may contribute to CGS's huge genome size. Obviously, we missed parts of genes, due to that we only sequenced tissues in adult period. Other developmental stages need complement in future study. In other hand, the present gene sets maybe include some non-coding genes or other noises, even if we used a most strict pipeline to identify the proteins. This is puzzle of RNA-seq data to identify coding genes. It needs to be verified by other data, full-length transcripts and protein data which ought to produce in the future. In addition, our strategy of de novo transcriptome assembly and protein identification works high effectively, and it is applicable to a wide range of other similar studies.

Availability of supporting data

All the clean reads were deposited in the National Center for Biotechnology Information (NCBI) and could be accessed in the Short Read Archive (SRA accession: SRP092015) linking to BioProject accession number PRJNA350354. The assemblies and annotations data and other relevant data have also been hosted in the *GigaScience* repository, GigaDB [17].

Acknowledgements

This work was supported by grants from the National Natural Science Foundation of China (No. 31572270), and the Major Scientific and Technological Projects of Henan (No. 111100910600). The funders had no role in study design, data

collection and analysis, decision to publish, or preparation of the manuscript.

Competing interests

The authors declare that they have no competing interests.

Authors' contributions

CSX, XDF and HW conceived the study and designed the experiments. HTS, QG, XYZ and JLG performed the experiments. WSL, XFG, JL and JBJ analyzed the data. BHZ, YW, BC, ZGQ, MHZ, FCZ, JM and JBJ contributed reagents/materials/analysis tools. XFG and WSL wrote the manuscript with input from all authors. CSX, HW, FCZ and JM revised the paper. All authors read and approved the final manuscript.

Additional files

Additional file 1: The expression profiling of all genes.

Reference

1. Gao KQ, Shubin NH. Earliest known crown-group salamanders. Nature. 2003;422:424-8.
2. Zhu R, Chen ZY, Wang J, Yuan JD, Liao XY, Gui JF, et al. Extensive diversification of MHC in Chinese giant salamanders Andrias davidianus (Anda-MHC) reveals novel splice variants. Dev Comp Immunol. 2014;42:311-22.
3. Zhu R, Chen ZY, Wang J, Yuan JD, Liao XY, Gui JF, et al. Thymus cDNA library survey uncovers novel features of immune molecules in Chinese giant salamander Andrias davidianus. Dev Comp Immunol. 2014;46:413-22.
4. Zhu B, Feng Z, Qu A, Gao H, Zhang Y, Sun D, et al. Brief report. The karyotype of the caudate amphibian Andrias davidianus. Hereditas. 2002;136:85-8.
5. Fan Y, Chang MX, Ma J, LaPatra SE, Hu YW, Huang L, et al. Transcriptomic analysis of the host response to an iridovirus infection in Chinese giant salamander, Andrias davidianus. Vet Res. 2015;46:136.

6. Li F, Wang L, Lan Q, Yang H, Li Y, Liu X, et al. RNA-Seq analysis and gene discovery of Andrias davidianus using Illumina short read sequencing. PLoS One. 2015;10:e0123730.

7. Qi Z, Zhang Q, Wang Z, Ma T, Zhou J, Holland JW, et al. Transcriptome analysis of the endangered Chinese giant salamander (Andrias davidianus): Immune modulation in response to Aeromonas hydrophila infection. Vet Immunol Immunopathol. 2016;169:85-95.

8. Grabherr MG, Haas BJ, Yassour M, Levin JZ, Thompson DA, Amit I, et al. Full-length transcriptome assembly from RNA-Seq data without a reference genome. Nat Biotechnol. 2011;29:644-52.

9. Simao FA, Waterhouse RM, Ioannidis P, Kriventseva EV, Zdobnov EM. BUSCO: assessing genome assembly and annotation completeness with single-copy orthologs. Bioinformatics. 2015;31:3210-2.

10. Li H, Coghlan A, Ruan J, Coin LJ, Heriche JK, Osmotherly L, et al. TreeFam: a curated database of phylogenetic trees of animal gene families. Nucleic Acids Res. 2006;34:D572-80.

11. Yu XJ, Zheng HK, Wang J, Wang W, Su B. Detecting lineage-specific adaptive evolution of brain-expressed genes in human using rhesus macaque as outgroup. Genomics. 2006;88:745-51.

12. Langmead B, Salzberg SL. Fast gapped-read alignment with Bowtie 2. Nat Methods. 2012;9:357-9.

13. Li B, Dewey CN. RSEM: accurate transcript quantification from RNA-Seq data with or without a reference genome. BMC Bioinformatics. 2011;12:323.

14. Mortazavi A, Williams BA, McCue K, Schaeffer L, Wold B. Mapping and quantifying mammalian transcriptomes by RNA-Seq. Nat Methods. 2008;5:621-8.

15. Sun C, Shepard DB, Chong RA, Lopez Arriaza J, Hall K, Castoe TA, et al. LTR retrotransposons contribute to genomic gigantism in plethodontid salamanders. Genome Biol Evol. 2012;4:168-83.

16. Sun C, Mueller RL. Hellbender genome sequences shed light on genomic expansion at the base of crown salamanders. Genome Biol Evol. 2014;6:1818-29.

17. Geng X, Li W, Shang H, Gou Q, Zhang F, Zang X, Zeng B, Li J, Wang Y, Ma J, Guo J, Jian J, Chen B, Qiao Z, Zhou M, Wei H, Fang X, Xu C: Supporting data for "A reference gene set construction

using RNA-seq of multiple tissues of Chinese Giant Salamander, Andrias davidianus" *GigaScience* Database. 2017. http://dx.doi.org/10.5524/100277

Tables

Table 1. Summary statistics of sequencing data and Q20 percentage.

Samples	Clean reads	Clean data	Q20 % of fq1	Q20 % of fq2
abdominal skin	71,388,238	6,424,941,420	97.86	97.15
blood	73,523,050	6,617,074,500	97.83	96.23
brain	72,150,562	6,493,550,580	98.10	97.24
cartilage	72,085,300	6,487,677,000	98.00	97.33
dorsal skin	71,852,996	6,466,769,640	97.82	96.91
eye	72,360,422	6,512,437,980	97.96	97.44
fat	72,747,654	6,547,288,860	97.01	95.82
fingertip	71,793,242	6,461,391,780	98.04	97.38
heart	71,465,342	6,431,880,780	98.00	96.71
kidney	73,287,452	6,595,870,680	98.00	96.31
lateral skin	71,640,046	6,447,604,140	97.95	97.30
liver	71,772,352	6,459,511,680	98.18	96.90
long bone	72,620,898	6,535,880,820	97.06	96.43
lung	73,629,864	6,626,687,760	97.86	96.46
maxillary	71,368,582	6,423,172,380	97.94	96.73
muscle	73,184,476	6,586,602,840	97.73	96.12
ovary	73,636,484	6,627,283,560	97.95	96.69
pancreas	71,963,574	6,476,721,660	97.21	96.51
skull	73,445,086	6,610,057,740	97.60	96.52
small intestine	71,451,888	6,430,669,920	97.40	96.63
spinal cord	72,208,398	6,498,755,820	98.14	97.30
spleen	71,432,332	6,428,909,880	97.37	96.59
stomach	73,740,532	6,636,647,880	97.96	96.56
tail fat	72,435,894	6,519,230,460	98.03	97.61

Table 2. The statistics of final assembly and coding gene prediction.

total data (Mb)	total length (bp)	total number (≥250bp)	total number (≥1kp)	average length	coding gene	non-coding genes
156,347	123,835,135	93,366	34,840	1,326	26,135	67,231

Table 3. Statistics for functional annotation.

Functional database	Number of sequences annotated
NR	41,043
Swiss-Prot	30,049
KEGG	30,528
COG	13,229
GO	16,369
Total	41,874

Table 4. The results of gene family classification.

species	total genes	un-clustered genes	gene families	unique families	average genes per family
A. davidianus	26,135(25,965)*	6,341	12,188	520	1.62
X.tropicalis	18,429	218	13,235	21	1.38
N.parkeri	22,972	2,391	13,986	306	1.47
A.carolinensis	17,767	818	13,387	30	1.27
P.sinensis	18,164	638	13,548	31	1.29
D.rerio	26,046	1,453	13,832	177	1.78
O.latipes	19,671	1,461	12,437	138	1.46
H.sapiens	21,375	2,062	15,542	409	1.24

Asterisk (*) represents gene number after correction.

Table 5. The statistics of transcripts and coding genes expressed in each samples.

samples	expressed transcripts	coding genes	samples	expressed transcripts	coding genes
abdominal skin	53,324	20,193	long bone	56,286	19,754
dorsal skin	60,446	21,580	lung	70,132	22,991
lateral skin	53,285	20,437	maxillary	59,431	21,424
blood	56,540	19,994	muscle	49,582	19,968
brain	66,923	22,715	ovary	53,343	21,072
cartilage	59,724	20,979	pancreas	44,177	18,746
eye	67,769	22,826	skull	59,933	22,206
fat	65,586	21,570	small intestine	59,156	21,588

fingertip	63,582	21,626	spinal cord	64,808	22,423
heart	62,127	21,734	spleen	64,258	21,699
kidney	66,223	22,792	stomach	58,688	21,601
liver	59,755	21,622	tail fat	63,090	21,264

Figure captions

Fig. 1. Huge RNA-seq data assembly. (A) The pipeline for de novo assembly, quality filter, and gene identification and classification. (B) The statistics of mapping rate before and after transcripts filter. Compared to total mapping ratio, the unique mapping rate was less than 2%, except sample 'stomach'. Moreover, the total mapping rate was a slight decrease in comparison to the result before filter.

Fig. 2. Identification and evaluation of giant salamander gene set. (A) The pipeline of prediction of coding genes. PRD represents Western clawed frog protein set, 947 proteins of CGS and 554 proteins of Newt from NCBI. (B) The results of BUSCO estimation. Asterisk (*) represents the final protein sets; pound ($^{\#}$) represents the primary protein sets. (C) Comparison of the length of homologous region to *X. tropicalis* and *N. parkeri*. The X-aixs is the ratio of length, and the Y-aixs is the percentage of gene number. (D) Comparison of the length of homologous sequence to *X. tropicalis* and *N. parkeri*. The X-aixs is log base 2 of length, and the Y-aixs is the percentage of gene number.

Fig. 3. Hierarchical clustering of gene expression profiling. Coding genes (left); non-coding genes (right). The coding genes have higher expression abundances than non-coding genes.

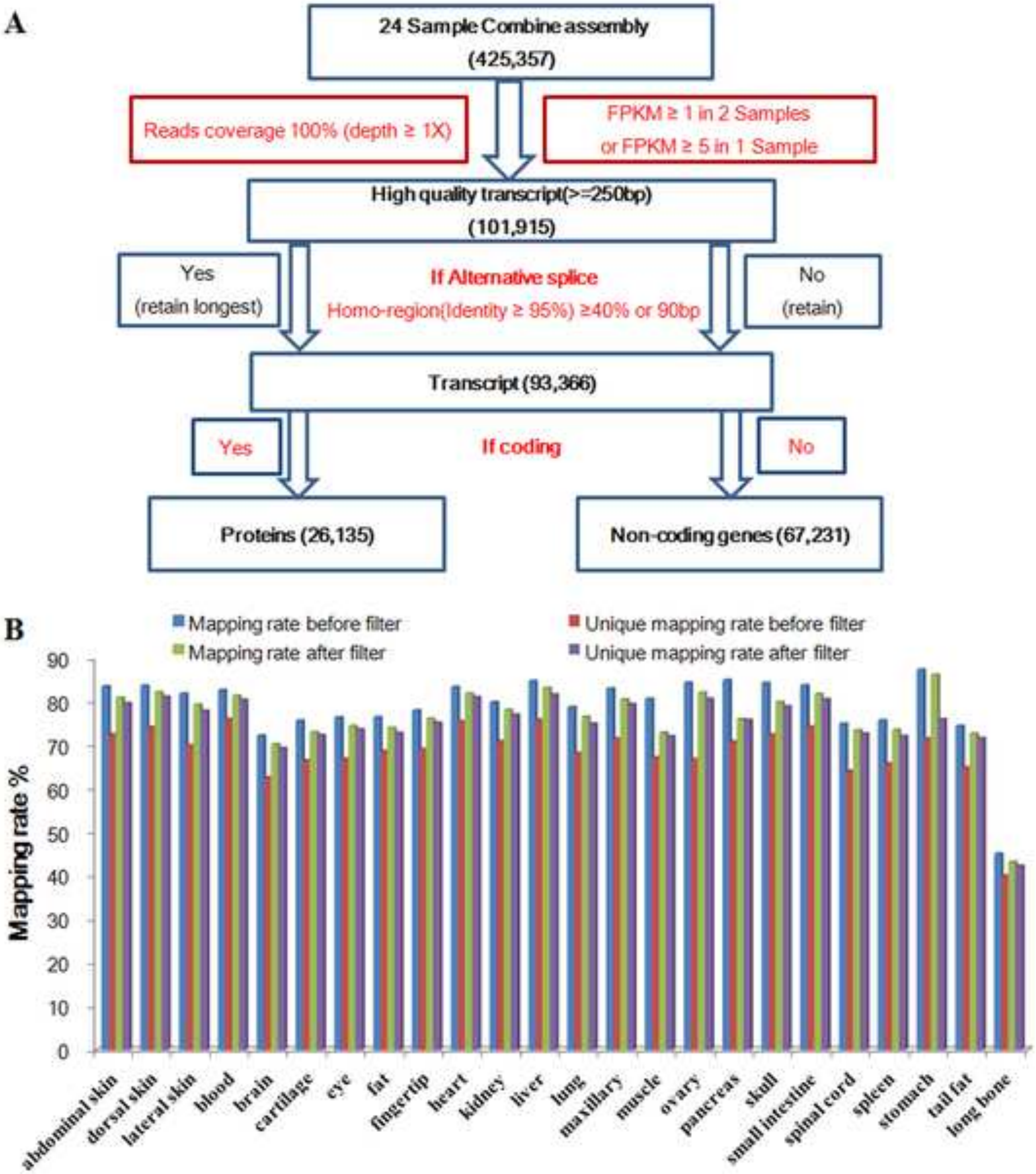
A
24 Sample Combine assembly
(425,357)
Reads coverage 100% (depth ≥ 1X)
FPKM ≥ 1 in 2 Samples
or FPKM ≥ 5 in 1 Sample
High quality transcript(>=250bp)
(101,915)
Yes
(retain longest)
If Alternative splice
Homo-region(Identity ≥ 95%) ≥40% or 90bp
No
(retain)
Transcript (93,366)
Yes
If coding
No
Proteins (26,135)
Non-coding genes (67,231)
B
Mapping rate before filter
Mapping rate after filter
Unique mapping rate before filter
Unique mapping rate after filter
Mapping rate %
0
10
20
30
40
50
60
70
80
90
abdominal skin
dorsal skin
lateral skin
blood
brain
cartilage
eye
fat
fingertip
heart
kidney
liver
lung
maxillary
muscle
ovary
pancreas
skull
small intestine
spinal cord
spleen
stomach
tail fat
long bone

Figure 2

Click here to download Figure Figure 2.tif

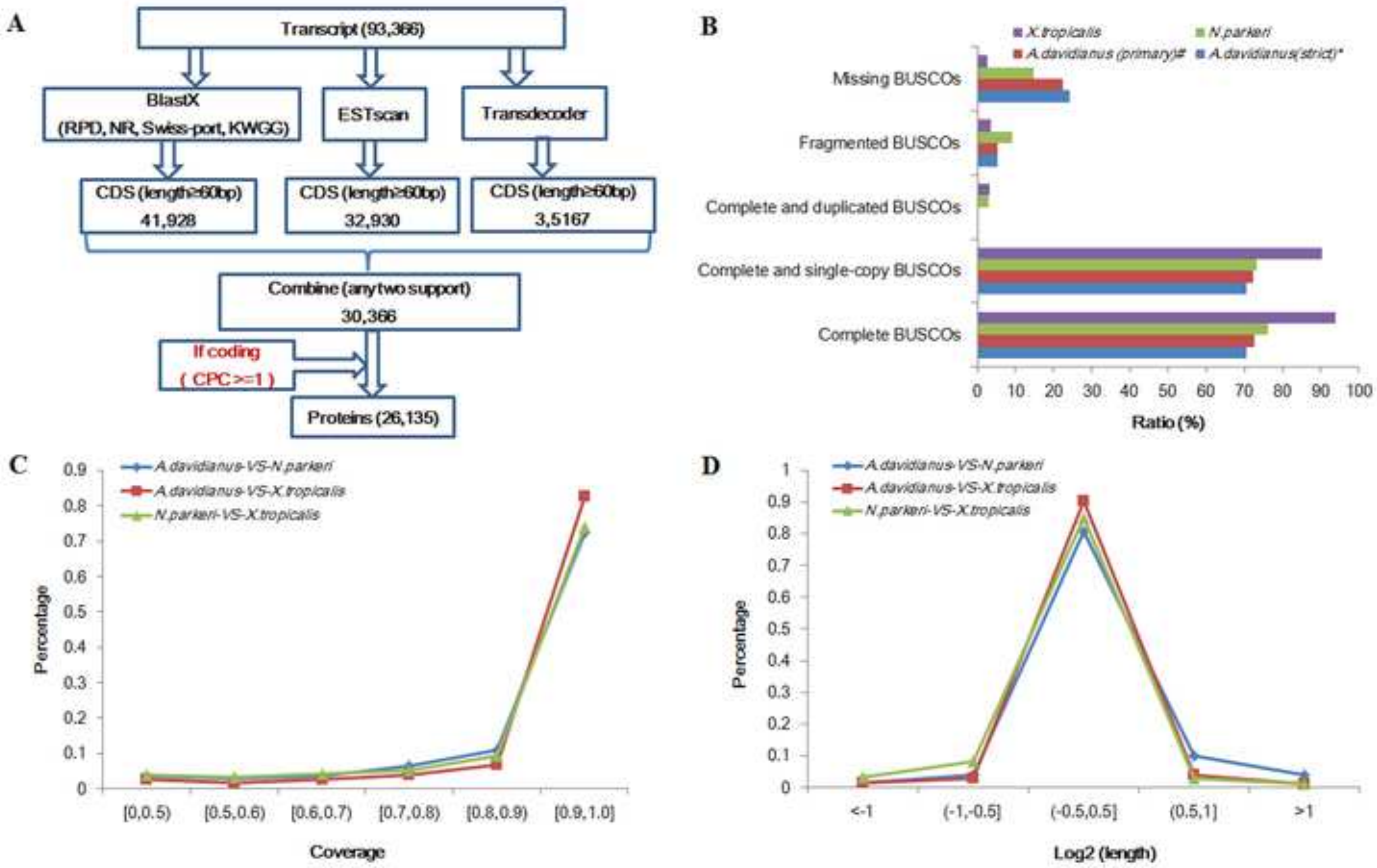

Figure 3

Click here to download Figure Figure 3.tif

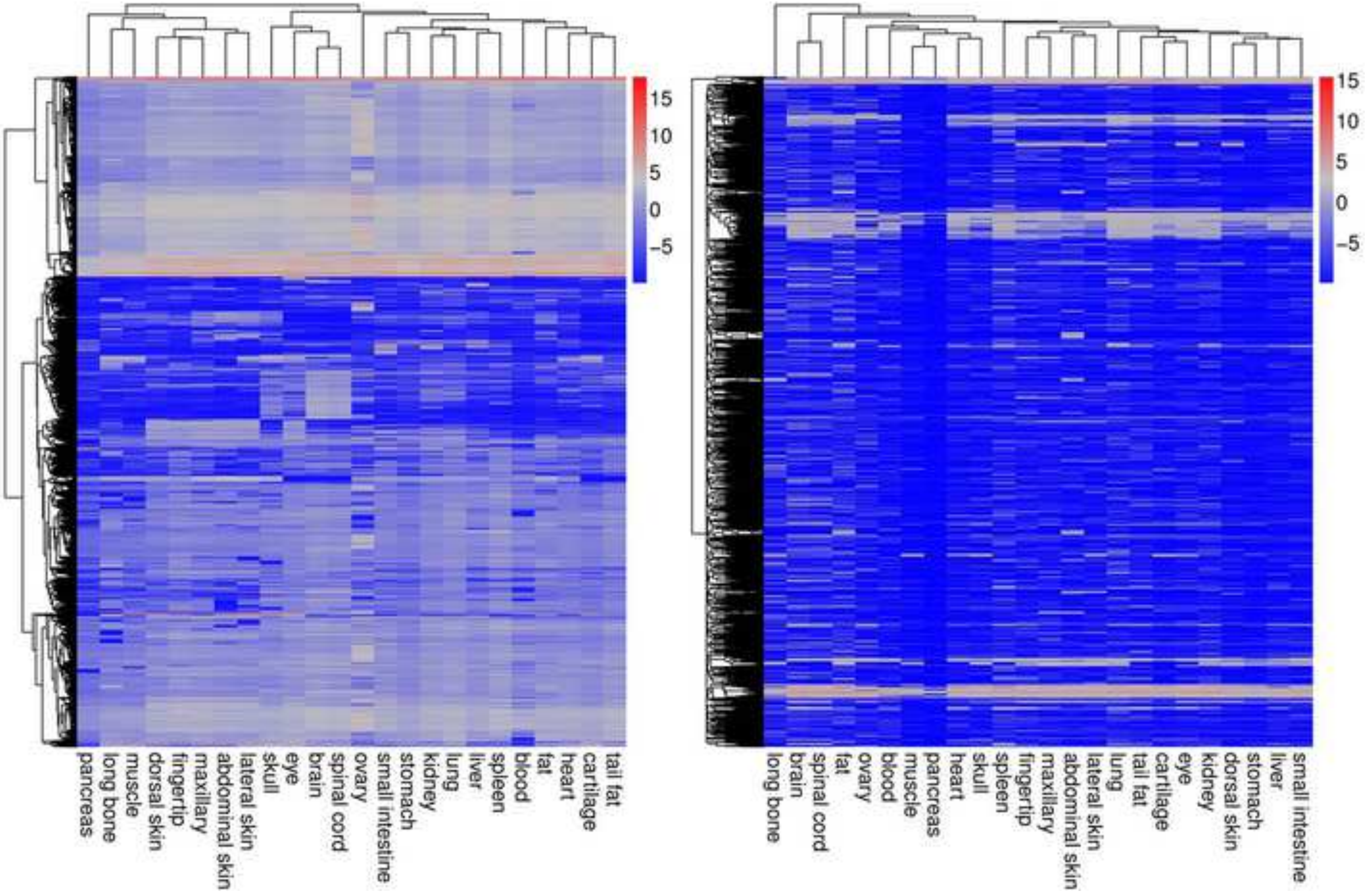

Click here to access/download
Supplementary Material
Additional file 1.xlsx

Personal Cover Click here to download Personal Cover Cover letter.doc

Dear Editor:

We would like to submit the manuscript “A reference gene set construction using RNA-seq of multiple tissues of Chinese Giant Salamander, *Andrias davidianus*” for publication in GigaScience as a "Data Note".

Chinese giant salamander (CGS) is the largest extant amphibian species in the world. Owing to its evolutionary position and four peculiar phenomenon of life (longevity, starvation tolerance, regenerative ability, and hatch without sunshine), it is an invaluable model species for research. However, lack of genomic resources leads to fewer study progresses in these field, due to its huge genome of ~50 GB extremely difficult to be sequenced. We reported the sequenced transcriptome of more than twenty tissues from adulthood of CGS using Illumina Hiseq 2000 technology, and a total of 93,366 no redundancy transcripts with a mean length of 1,326 bp were obtained. We for the first time developed an efficient pipeline to construct a high quality reference gene set of CGS and obtained 26,135 coding genes. BUSCO and homologous assessment showed that our assembly captured 70.6 % of vertebrate universal single-copy orthologs, and this coding gene set had a higher proportion of completeness CDS with comparable quality of the protein sets of Tibetan frog. These highest quality data will provide valuable reference gene set to the subsequent research of CGS. In addition, our strategy of de novo transcriptome assembly and protein identification is applicable to similar studies.

Above all, we strongly feel that this work is indeed suitable for the broad scientific audience of your journal.

Many thanks in advance for your time and considerations.

Sincerely yours,

Dr. Xiaofang Geng

Corresponding authors: Prof. Cunshuan Xu (cellkeylab@126.com); Prof. Xiaodong Fang (fangxd@genomics.cn); Prof. Hong Wei (weihong63528@163.com)
