# Supplementary material for: A reference gene set construction using RNA-seq of multiple tissues of Chinese giant salamander, Andrias davidianus
Source: Gigascience. 2017 Feb 15;6(3):1–7. doi: 10.1093/gigascience/gix006 (PMC5467019; doi:10.1093/gigascience/gix006)
Supplement: Response_to_Reviewer_Comment_Original_Submission.pdf [file gix006_response_to_reviewer_comment_original_submission.pdf]

Respose to Editor
1. If you revise the paper as a "Data Note", more summary statistics for the gene set would be desirable and a detailed description of your transcriptome assembly pipeline should get special attention
Answer: Thanks for your advice. We decide to revise this paper as a "Data Note". According to your suggestion, more summary statistics for the gene set and a detailed description of our transcriptome assembly pipeline can be seen in the revised manuscript.
Respose to Reviewers
Reviewer #1: In this manuscript, Xiaofang Geng and colleagues reported the gene set of Chinese giant salamander (CGS; Andrias davidianus), using massive transcriptome data collected from 24 different tissues. As the authors mentioned, because of many interesting characteristics that this organism has, such as longevity and starvation tolerance, and its difficulty to conduct whole genome sequencing due to gigantic genome size (~ 50 GB), this gene set would be definitely useful resource for other researchers in the future. I also agree that the quality of assembled transcripts is quite good, comparable to other published amphibian gene set. Considering the value of raw transcriptome data of 24 different CGS tissues and the gene set that the authors reported, I think this manuscript is worth to be published. However, as a 'Research Article', the analysis related to longevity and starvation tolerance were not solid enough to convince the readers (see below for more detailed reasons). If authors want to reshape this as a 'Data Note', it would be better focused on the transcriptome assembly pipeline and gene set evaluation step instead of biological analysis of CGS-specific genes.
Answer: Thanks for your advice. We have revised this paper as a "Data Note". We focus more on the transcriptome assembly pipeline and gene set evaluation step which can be seen in the revised manuscript.
1. CGS-specific genes
The authors claimed that there are 3,839 CGS-specific genes, and concluded them these genes are involved in KEGG pathways related to longevity and starvation tolerance. However, in my opinion, the authors' logic is not clear to make that conclusion.
- In the method described on page 22 last paragraph, what is the rationale to compare newt and X. tropicalis transcripts? Newt transcripts are incomplete, and X. tropicalis proteins are already compared in previous step, so I cannot understand how extra genes are filtered out here. Also, it should be corrected that whether the authors used "BLASTP or BLASTX" for proteins, or "BLASTN" for transcripts ("BLASTN" cannot use on proteins).
- Next step is much more confused. Authors identified "CGS-specific genes" which do not have homolog candidates in other species. But there is no CGS record on KEGG pathway. Then, how can the authors assign these "CGS-specific genes" to each KEGG pathway computationally?
- In the section for 'longevity' and 'starvation tolerance', authors just listed up the pathways that CGS-specific genes may be involved. However, there is no statistical test to estimate their significance here. Just one gene in insulin signaling pathway (page 8, line 19-20) may not be enough to claim that CGS has specific insulin signaling pathway. Authors also need to bring statistical method to estimate the significance in other parts of manuscript (there are several places authors mentioned 'significance', but I cannot find what kind of statistical test they used to estimate that).
Answer: Thanks for your advice. We have revised this paper as a "Data Note". According to your suggestion, we focus on the transcriptome assembly pipeline and gene set evaluation step in the revised manuscript. Therefore, we deleted the analytical contents of CGS-specific genes for there exist ambiguity in the identification of CGS-specific genes.
2. Gene expression analysis
In the 'characteristic of each tissue' section, authors performed co-expression clustering analysis and described the members on each module. However, I cannot see any new findings there. It is already well known that genes in the same pathway show co-expression patterns in multiple conditions generally. If authors found something interesting, especially related to longevity and starvation tolerance, the detail should be described here.
- Differential gene expression among three skins are not obviously detectable in Fig 5F (they all look quite similar). Also, to claim their differential expression, the negative control (which does not change, and how many genes are categorized in this 'non-DE genes') should be presented. Without multiple replicates, it is hard to judge how significantly they are different.
- Please don't refer co-expression modules as their 'colors'. If you can find a tissue specificity, or any other

character that you can define each module, please use those 'biologically meaningful' names.
Answer: Thanks for your advice. We have revised this paper as a "Data Note". Therefore, we deleted the analytical contents of “characteristic of each tissue”, “differential gene expression among three skins” and co-expression modules for there no new findings in these contents.
3. - Page 6 line 5. Hierarchical clustering part is not suitable for this section. Please rearrange it to gene expression analysis part.
- Page 13 line 16-18. Is this animal infected by V. cholerae?
- All figures are blurred so I could not read any letter like Fig 4. Please correct them.
- I could not find any point for 'hairy-ball' Figure 5, because I cannot see any letter on it. If you want to emphasize something here, it would be better to re-draw them selectively.
Answer: Thank you for your suggestion. We have rearrangeed Hierarchical clustering to gene expression analysis part. Additionally, the revised manuscript did not contain the contents of Figure 4 and Figure 5, so we deteleted them.
Reviewer #2: Geng et al. present data on a transcriptome assembly of the Chinese giant salamander, Andrias davidianus, which is a model species for studies of longevity and starvation tolerance. In addition, the secretions of this salamander have a role as a traditional Chinese medicine, making its gene products of commercial interest. The authors have adapted standard workflows to identify 26,135 coding genes from analysis of 24 distinct tissues.
While extensive text is devoted to description of the GO and KEGG categories for the differentially expressed genes, the main finding of this study is the transcriptome assembly and may be better served through the format of a more concise data note. Association of gene expression in this one species is not equivalent to causality, and identification of genes unique to the long-lived A. davidianus may not necessarily be critical in the extended lifespan of this organism. As such, this decreases confidence in the impact of associative data presented in Figures 3-5. Condensing the findings to focus on the orthology assignments for A. davidianus novel genes may be more informative. Given the limited availability of gene sequences from amphibians, due to large genome sizes, the availability of the A. davidianus transcriptome assembly is a welcome addition for comparative genomic studies.
Answer: Thanks for your advice. We have revised this paper as a "Data Note". We focus on more summary statistics for the gene set, a detailed description of our transcriptome assembly pipeline and gene set evaluation which can be seen in the revised manuscript.

Sincerely yours,
Dr. Xiaofang Geng
Corresponding authors: Prof. Cunshuan Xu (cellkeylab@126.com); Prof. Xiaodong Fang (fangxd@genomics.cn); Prof. Hong Wei (weihong63528@163.com)
