# Supplementary material for: A reference gene set construction using RNA-seq of multiple tissues of Chinese giant salamander, Andrias davidianus
Source: Gigascience. 2017 Feb 15;6(3):1–7. doi: 10.1093/gigascience/gix006 (PMC5467019; doi:10.1093/gigascience/gix006)
Supplement: Reviewer_1_Report_(Original_Submission).pdf [file gix006_reviewer_1_report_(original_submission).pdf]

Reviewer Report

Title: "A reference gene set construction using RNA-seq of multiple tissues of Chinese Giant Salamander, Andrias davidianus"

Version: Original Submission **Date:** 12/6/2016

Reviewer name: Taejoon Kwon

Reviewer Comments to Author:

In this manuscript, Xiaofang Geng and colleagues reported the gene set of Chinese giant salamander (CGS; Andrias davidianus), using massive transcriptome data collected from 24 different tissues. As the authors mentioned, because of many interesting characteristics that this organism has, such as longevity and starvation tolerance, and its difficulty to conduct whole genome sequencing due to gigantic genome size (~ 50 GB), this gene set would be definitely useful resource for other researchers in the future. I also agree that the quality of assembled transcripts is quite good, comparable to other published amphibian gene set. Considering the value of raw transcriptome data of 24 different CGS tissues and the gene set that the authors reported, I think this manuscript is worth to be published. However, as a 'Research Article', the analysis related to longevity and starvation tolerance were not solid enough to convince the readers (see below for more detailed reasons). If authors want to reshape this as a 'Data Note', it would be better focused on the transcriptome assembly pipeline and gene set evaluation step instead of biological analysis of CGS-specific genes.

[Major issues]

1. CGS-specific genes

The authors claimed that there are 3,839 CGS-specific genes, and concluded them these genes are involved in KEGG pathways related to longevity and starvation tolerance. However, in my opinion, the authors' logic is not clear to make that conclusion.

- In the method described on page 22 last paragraph, what is the rationale to compare newt and X. tropicalis transcripts? Newt transcripts are incomplete, and X. tropicalis proteins are already compared in previous step, so I cannot understand how extra genes are filtered out here. Also, it should be corrected that whether the authors used "BLASTP or BLASTX" for proteins, or "BLASTN" for transcripts ("BLASTN" cannot use on proteins).

- Next step is much more confused. Authors identified "CGS-specific genes" which do not have homolog candidates in other species. But there is no CGS record on KEGG pathway. Then, how can the authors assign these "CGS-specific genes" to each KEGG pathway computationally?

- In the section for 'longevity' and 'starvation tolerance', authors just listed up the pathways that CGS-specific genes may be involved. However, there is no statistical test to estimate their significance here. Just one gene in insulin signaling pathway (page 8, line 19-20) may not be enough to claim that CGS has specific insulin signaling pathway. Authors also need to bring statistical method to estimate the significance in other parts of manuscript (there are several places authors mentioned 'significance', but I cannot find what kind of statistical test they used to estimate that).

2. Gene expression analysis

- In the 'characteristic of each tissue' section, authors performed co-expression clustering analysis and described the members on each module. However, I cannot see any new findings there. It is already well known that genes in the same pathway show co-expression patterns in multiple conditions generally. If authors found something interesting, especially related to longevity and starvation tolerance, the detail should be described here.

- Differential gene expression among three skins are not obviously detectable in Fig 5F (they all look quite similar). Also, to claim their differential expression, the negative control (which does not change, and how many genes are categorized in this 'non-DE genes') should be presented. Without multiple replicates, it is hard to judge how significantly they are different.

- Please don't refer co-expression modules as their 'colors'. If you can find a tissue specificity, or any other character that you can define each module, please use those 'biologically meaningful' names.

[Minor issues]

- Page 6 line 5. Hierarchical clustering part is not suitable for this section. Please rearrange it to gene expression analysis part.

- Page 13 line 16-18. Is this animal infected by V. cholerae?

- All figures are blurred so I could not read any letter like Fig 4. Please correct them.

- I could not find any point for 'hairy-ball' Figure 5, because I cannot see any letter on it. If you want to emphasize something here, it would be better to re-draw them selectively.

Methods

Are the methods appropriate to the aims of the study, are they well described, and are necessary controls included? No

Conclusions

Are the conclusions adequately supported by the data shown? Yes

Reporting Standards

Does the manuscript adhere to the journal's guidelines on minimum standards of reporting? No

Statistics

Are you able to assess all statistics in the manuscript, including the appropriateness of statistical tests used? Yes, and I have assessed the statistics in my report.

Quality of Written English

Please indicate the quality of language in the manuscript: Needs some language corrections before being published

Declaration of Competing Interests

Please complete a declaration of competing interests, considering the following questions:

- Have you in the past five years received reimbursements, fees, funding, or salary from an organisation that may in any way gain or lose financially from the publication of this manuscript, either now or in the future?
- Do you hold any stocks or shares in an organisation that may in any way gain or lose financially from the publication of this manuscript, either now or in the future?
- Do you hold or are you currently applying for any patents relating to the content of the manuscript?

- Have you received reimbursements, fees, funding, or salary from an organization that holds or has applied for patents relating to the content of the manuscript?
- Do you have any other financial competing interests?
- Do you have any non-financial competing interests in relation to this paper?

If you can answer no to all of the above, write 'I declare that I have no competing interests' below. If your reply is yes to any, please give details below.

I declare that I have no competing interests

I agree to the open peer review policy of the journal. I understand that my name will be included on my report to the authors and, if the manuscript is accepted for publication, my named report including any attachments I upload will be posted on the website along with the authors' responses. I agree for my report to be made available under an Open Access Creative Commons CC-BY license (http://creativecommons.org/licenses/by/4.0/). I understand that any comments which I do not wish to be included in my named report can be included as confidential comments to the editors, which will not be published.

I agree to the open peer review policy of the journal

To further support our reviewers, we have joined with Publons, where you can gain additional credit to further highlight your hard work (see: https://publons.com/journal/530/gigascience). On publication of this paper, your review will be automatically added to Publons, you can then choose whether or not to claim your Publons credit. I understand this statement.

Yes
