# Supplementary material for: A reference gene set construction using RNA-seq of multiple tissues of Chinese giant salamander, Andrias davidianus
Source: Gigascience. 2017 Feb 15;6(3):1–7. doi: 10.1093/gigascience/gix006 (PMC5467019; doi:10.1093/gigascience/gix006)
Supplement: Reviewer_2_Report_(Original_Submission).pdf [file gix006_reviewer_2_report_(original_submission).pdf]

Reviewer Report

Title: "A reference gene set construction using RNA-seq of multiple tissues of Chinese Giant Salamander, Andrias davidianus"

Version: Original Submission **Date:** 12/7/2016

Reviewer name: Kenro Kusumi, PhD

Reviewer Comments to Author:

Geng et al. present data on a transcriptome assembly of the Chinese giant salamander, Andrias davidianus, which is a model species for studies of longevity and starvation tolerance. In addition, the secretions of this salamander have a role as a traditional Chinese medicine, making its gene products of commercial interest. The authors have adapted standard workflows to identify 26,135 coding genes from analysis of 24 distinct tissues.

While extensive text is devoted to description of the GO and KEGG categories for the differentially expressed genes, the main finding of this study is the transcriptome assembly and may be better served through the format of a more concise data note. Association of gene expression in this one species is not equivalent to causality, and identification of genes unique to the long-lived A. davidianus may not necessarily be critical in the extended lifespan of this organism. As such, this decreases confidence in the impact of associative data presented in Figures 3-5. Condensing the findings to focus on the orthology assignments for A. davidianus novel genes may be more informative.

Given the limited availability of gene sequences from amphibians, due to large genome sizes, the availability of the A. davidianus transcriptome assembly is a welcome addition for comparative genomic studies.

Methods

Are the methods appropriate to the aims of the study, are they well described, and are necessary controls included? Yes

Conclusions

Are the conclusions adequately supported by the data shown? No

Reporting Standards

Does the manuscript adhere to the journal's guidelines on minimum standards of reporting? Yes

Statistics

Are you able to assess all statistics in the manuscript, including the appropriateness of statistical tests used? Yes, and I have assessed the statistics in my report.

Quality of Written English

Please indicate the quality of language in the manuscript: Not suitable for publication unless extensively edited

Declaration of Competing Interests

Please complete a declaration of competing interests, considering the following questions:

- Have you in the past five years received reimbursements, fees, funding, or salary from an organisation that may in any way gain or lose financially from the publication of this manuscript, either now or in the future?
- Do you hold any stocks or shares in an organisation that may in any way gain or lose financially from the publication of this manuscript, either now or in the future?
- Do you hold or are you currently applying for any patents relating to the content of the manuscript?
- Have you received reimbursements, fees, funding, or salary from an organization that holds or has applied for patents relating to the content of the manuscript?
- Do you have any other financial competing interests?
- Do you have any non-financial competing interests in relation to this paper?

If you can answer no to all of the above, write 'I declare that I have no competing interests' below. If your reply is yes to any, please give details below.

I declare that I have no competing interests.

I agree to the open peer review policy of the journal. I understand that my name will be included on my report to the authors and, if the manuscript is accepted for publication, my named report including any attachments I upload will be posted on the website along with the authors' responses. I agree for my report to be made available under an Open Access Creative Commons CC-BY license (http://creativecommons.org/licenses/by/4.0/). I understand that any comments which I do not wish to be included in my named report can be included as confidential comments to the editors, which will not be published.

I agree to the open peer review policy of the journal

To further support our reviewers, we have joined with Publons, where you can gain additional credit to further highlight your hard work (see: https://publons.com/journal/530/gigascience). On publication of this paper, your review will be automatically added to Publons, you can then choose whether or not to claim your Publons credit. I understand this statement.

Yes
